# Increased risk of arrhythmias, heart failure, and thrombosis in SARS-CoV-2 positive individuals persists at one year post-infection

**DOI:** 10.1016/j.csbj.2024.06.024

**Published:** 2024-06-20

**Authors:** C. Tintore, J. Cuartero, A. Camps-Vilaró, Subirana, R. Elosua, J. Marrugat, IR Degano

**Affiliations:** a Faculty of Medicine, University of Vic-Central University of Catalonia, 08500 Vic, Spain; b Department of Medicine, Universitat Autònoma de Barcelona, 08193 Barcelona, Spain; c Department of Oncology, Hospital de la Santa Creu i Sant Pau, 08041 Barcelona, Spain; d Centro de Investigación Biomédica en Red de Enfermedades Cardiovasculares (CIBERCV), Instituto de Salud Carlos III, 28029 Madrid, Spain; e Registre Gironí del Cor (REGICOR) Study Group, Hospital del Mar Medical Research Institute (IMIM), 08003 Barcelona, Spain; f Cardiovascular Epidemiology and Genetics Research Group, IMIM, 08003 Barcelona, Spain; g Institute for Research and Innovation in Life Sciences and Health in Central Catalonia (IRIS-CC), 08500 Vic, Spain

**Keywords:** SARS-CoV-2, COVID-19, Cardiovascular disease, Mortality

## Abstract

Risk of cardiovascular events is increased after COVID-19. However, information on cardiovascular risk trends after COVID-19 infection is lacking and estimates by sex are inconsistent. Our aim was to examine cardiovascular outcomes and mortality in a large cohort (164,346 participants) of SARS-CoV-2 positive individuals compared to non-positive individuals, stratified by sex. Data were obtained from the Spanish Health System’s electronic medical records. Selected individuals were ≥ 45 years old with/without a positive SARS-CoV-2 test in the period March-May 2020. Follow-up was obtained until January 31, 2021, for cardiovascular events (angina/myocardial infarction, arrhythmias, bypass/revascularization, heart failure, peripheral artery disease, stroke/transient ischemic attack, and thrombosis), and until March 31, 2021, for mortality. Individuals were matched by propensity score. Incidence of cardiovascular events and mortality was compared with accelerated failure time models. The effect of matching and of COVID-19 severity was assessed with sensitivity analyses. In the first 3 months of follow-up, SARS-CoV-2 positive individuals had a higher risk of mortality and of all cardiovascular events. From 4–12 months, there was increased risk of mortality in SARS-CoV-2 positive individuals overall, of heart failure in SARS-CoV-2 positive females (HR= 1.26 [1.11–1.42]), and of arrhythmias and thrombosis in SARS-CoV-2 positive males (HR= 1.29 [1.14–1.47] and HR= 1.35 [1.03–1.77], respectively). When COVID-19 patients admitted to the ICU were excluded, incidence of thrombosis was similar in males regardless of positive/non-positive SARS-CoV-2 status. In the full year of follow-up, increased incidence of heart failure and of arrhythmias and thrombosis was observed in SARS-CoV-2 positive females and males, respectively.

## Highlights

• During the first 3 months after SARS-CoV-2 infection.
• There is an increased risk of CVD events including bypass/revascularization and peripheral artery disease.
• The events showing the highest risk in SARS-CoV-2 positive individuals are arrhythmias, coronary events and thrombosis.
• 4-12 months after SARS-CoV-2 positivity.
• There is an increased risk of heart failure in females, and of arrhythmias and thrombosis in males.

## Graphical Abstract

**Figure:**
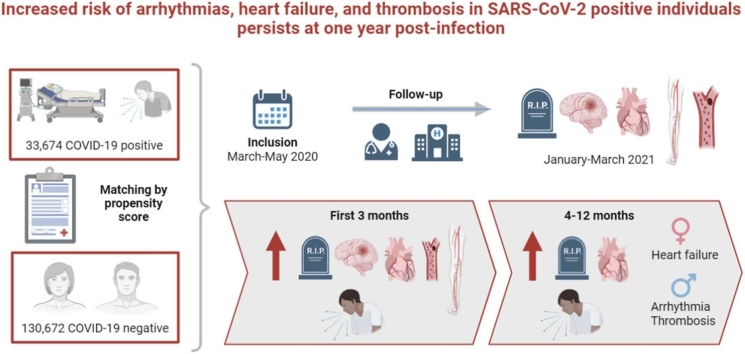

## 1 Introduction

Severe acute respiratory syndrome coronavirus 2 (SARS-CoV-2) or coronavirus disease 19 (COVID-19) initiates a respiratory infection that can progress to pneumonia and acute respiratory distress syndrome [1]. In addition, SARS-CoV-2 infection can also trigger a cytokine storm and systemic inflammation, potentially leading to multiorgan damage and coagulation abnormalities [2]. In turn, anomalies in coagulation increase the risk of thromboembolic events [3], [4].

Early studies of COVID-19 highlighted its association with cardiovascular disease (CVD) during the acute phase of infection. In hospitalized COVID-19 patients, prevalence of CVD (especially coronary heart disease) and of CVD risk factors (especially hypertension and diabetes) ranged from 10 % to 30 % [5], [6], [7]. Moreover, the presence of CVD and its risk factors correlated with increased in-hospital mortality among COVID-19 patients [8], [9]. Acute manifestations of CVD such as acute coronary syndrome, arrhythmias, heart failure, and thrombosis were also observed among hospitalized COVID-19 patients [9], [10], [11].

Recent large-scale studies examining CVD outcomes following SARS-CoV-2 infection or COVID-19 diagnosis, with follow-up periods ranging from 9 months to 2 years, have indicated an elevated risk of CVD events among those in contact with the SARS-CoV-2 virus, compared to control individuals [12], [13], [14], [15], [16], [17], [18], [19]; however, the specific CVD outcomes presenting the highest risk remain unclear. Furthermore, most studies have excluded the initial 21–30 days of follow-up, but none has explored whether CVD incidence remains increased during different periods of the follow-up. On the other hand, only 3 of the published studies have stratified their analysis by sex, yielding conflicting results [12], [13], [16]. Additionally, limited data are available on the influence of COVID-19 severity on the association between SARS-CoV-2 and CVD incidence.

Our aim was to conduct a matched analysis, in a large cohort drawn from electronic medical records, to assess the incidence of CVD events and mortality among SARS-CoV-2 positive versus non-positive individuals during the first 3 months post-infection and from the fourth month to 1 year of follow-up. Secondary objectives included sex-stratified analysis, as well as a sensitivity analysis excluding severe COVID-19 patients.

## 2 Methods

### 2.1 Study design and data

This was a retrospective cohort study of general population from Catalonia, northeastern Spain. Data were obtained from the Data analytics program for health research and innovation (PADRIS), managed by the Health Quality and Assessment Agency of Catalonia (AQUAS). PADRIS aggregates data from various sources such as the Catalan Health Institute (CHI), a public healthcare system that covers 7.9 million inhabitants. PADRIS contains also information from admissions at public hospitals, epidemiological registries, pharmacy retrievals, the CHI’s primary care system, and the mortality register, among others [20]. However, PADRIS does not include data from private health services.

### 2.2 Population

We included individuals aged 45 years and older who tested positive for SARS-CoV-2 through polymerase chain reaction, antigen, or rapid tests between March 1, 2020, and May 31, 2020 (Fig. S1 in SI). We also included randomly selected individuals from the same age range who did not test positive for SARS-CoV-2. We excluded individuals with prior CVD, patients without positive SARS-CoV-2 tests that flagged as suspicious in a population-based COVID-19 registry, and individuals exhibiting extreme anthropometric or biochemical values (Table S1 in SI). The definition of prior CVD encompassed any of the following: angina, arrhythmias, bypass, heart failure, myocardial infarction, peripheral artery disease, revascularization, stroke, tachycardia, thrombosis and transient ischemic attack. Arrhythmias included atrial fibrillation, flutter, and tachycardia.

### 2.3 Baseline data

We retrieved data on risk factors from primary care and hospital admission records from March 1, 2017, to March 1, 2020. The most recent risk factor information prior to inclusion in the study was selected.

The recorded data included demographics (age and sex), details of SARS-CoV-2 tests (date and type of test), information on COVID-19 hospitalization (dates, length of hospitalization, intensive care unit (ICU) admission, intubation, and mechanical ventilation), and anthropometrics (body mass index, height, and weight). Hospitalization exceeding 1 day and recorded as due to COVID-19 was considered as a hospital stay. Body mass index was calculated as weight in kilograms divided by squared height in meters. Information was also collected on CVD risk factors (creatinine, glucose, high-density lipoprotein cholesterol, low-density lipoprotein cholesterol, total cholesterol, triglycerides, diastolic blood pressure, systolic blood pressure, and diagnosis and treatment of diabetes, hypercholesterolemia, and hypertension). Moreover, data on previous medical history of other conditions were obtained (cancer, dementia, liver failure, chronic obstructive pulmonary disease, and renal failure). Liver failure encompassed chronic hepatitis, fibrosis, cirrhosis, and necrosis. Diabetes, hypercholesterolemia, and hypertension were classified as positive if individuals had a prior diagnosis, were or had been receiving treatment for the condition, or if recorded levels met or exceeded a defined threshold: 126 mg/dL for glucose, 140/90 mm Hg for systolic/diastolic blood pressure, and 240 mg/dL for total cholesterol.

### 2.4 Follow-up

Follow-up data were obtained from hospital admissions records for CVD events until January 31, 2021, and from the mortality register until March 31, 2021. The collected CVD events during the follow-up included arrhythmias, bypass/revascularization, cerebrovascular events (stroke/transient ischemic attack), coronary events (angina/myocardial infarction), heart failure, mortality, peripheral artery disease, and thrombosis.

Risk factor and follow-up data were obtained using international classification of diseases (ICD) codes; treatment data were gathered using Anatomical Therapeutic Chemical (ATC) codes (Tables S2 and S3 in SI).

### 2.5 Ethics

The project was approved by the ethics committees of the *Hospital del Mar Research Institute* (IMIM) and PADRIS. The data used in this project were anonymized.

### 2.6 Statistical analysis

#### 2.6.1 Matching

We matched SARS-CoV-2 positive and non-positive individuals with the MatchIT package from R [21], using nearest neighbor matching with a 4:1 ratio and a caliper of 0.2. Matching was based on a logistic propensity score model for SARS-CoV-2 positivity, incorporating variables such as age, sex, smoking history, and previous medical conditions including diabetes, hypertension, hypercholesterolemia, cancer, chronic obstructive pulmonary disease, and liver and renal insufficiency. Matching quality was evaluated using the adjusted standardized mean difference [22], [23] as well as balance graphics. Balance graphics were obtained with the PSAgraphics R package. We created 5 stratum of similar size according to the propensity score values and analyzed the covariate mean or the covariate categories proportion in each stratum. After matching, all variables included in the propensity score model exhibited adjusted standardized mean differences < 0.1 and were well balanced in each PS stratum (Table S4 and Figs. S2-S11 in SI).

#### 2.6.2 Sample size

With a matched sample size of 164,346 individuals (33,674 [20.5 %] positive for SARS-CoV-2), the observed event rates in SARS-CoV-2 non-positive individuals (Table 1), and assuming an alpha risk of 0.05 and a beta risk of 0.2 in a two-sided test, we estimated the minimum hazard ratio (HR) that could be detected as statistically significant using the Poisson approximation. The minimum HRs were as follows: 1.15 for mortality, 1.25 for arrhythmias and heart failure, 1.40 for peripheral artery disease and stroke/transient ischemic attack, 1.45 for angina/myocardial infarction, 1.65 for bypass/revascularization, and 1.70 for thrombosis.

**Table 1:** Crude incidence of cardiovascular outcomes and mortality during the 12-month follow-up in the matched population by positivity for SARS-CoV-2.

	SARS-CoV-2 non-positive n = 130,672	SARS-CoV-2 positive n = 33,674	p-value
**Arrhythmias (AF/flutter/tachycardia)**	905 (0.71 %)	1155 (3.51 %)	< 0.001
**Bypass/revascularization**	114 (0.09 %)	107 (0.33 %)	< 0.001
**Cerebrovascular events (stroke/TIA)**	302 (0.24 %)	173 (0.53 %)	< 0.001
**Coronary events (angina/MI)**	238 (0.19 %)	116 (0.35 %)	< 0.001
**Heart failure**	736 (0.57 %)	592 (1.80 %)	< 0.001
**Mortality**	3055 (2.14 %)	4051 (13.1 %)	< 0.001
**Peripheral artery disease**	256 (0.21 %)	244 (0.74 %)	< 0.001
**Thrombosis**	84 (0.07 %)	256 (0.78 %)	< 0.001

Variables were compared between groups with the Chi-squared/Fisher exact tests. AF: atrial fibrillation; MI: myocardial infarction; SARS-CoV-2: severe acute respiratory syndrome coronavirus; TIA: transient ischemic attack.

#### 2.6.3 Descriptive analysis

The descriptive analysis encompassed demographic variables, COVID-19 hospitalization information, anthropometric measurements, CVD risk factors, previous medical history, and incidence of CVD events and mortality. Quantitative variables were described with the median and the 95 % confidence interval (CI), and compared with the Mann-Whitney U test, as they did not follow a normal distribution. Categorical variables were presented as absolute and relative frequencies and compared with the Chi-squared or Fisher exact test.

#### 2.6.4 Outcome incidence in the follow-up

Incidence of CVD events and mortality during the follow-up were compared between SARS-CoV-2 positive and non-positive individuals with accelerated failure time models. For each model, we selected the distribution that yielded the lowest Akaike Information Criterion. We plotted the model residuals and the follow-up time and verified that residuals were randomly distributed. Models accounted for matching weights and pairs, and cluster-robust standard errors were computed. Crude and adjusted models were fitted for the entire cohort and for females and males separately, considering the 12-month follow-up, the initial 3 months, and the period from the fourth month to the end of follow-up. We defined these periods because the steepest increase in the incidence of CVD outcomes took place during the first 3 months of the follow-up. Adjusted models included the covariates exhibiting statistical differences in the descriptive analysis: age, blood pressure (diastolic and systolic), body mass index, cholesterol (high density lipoprotein and total), and previous history of cancer, chronic obstructive pulmonary disease, dementia, hypercholesterolemia, liver and renal insufficiency. Crude and adjusted HRs and their 95 % CIs were calculated. HRs and CIs were obtained by multiplying the group model coefficient and the coefficient CIs by − 1 *shape parameter. The shape parameter was 1/scale parameter of the model.

#### 2.6.5 Sensitivity analyses

Two sensitivity analyses were performed. One included all individuals without matching and the other excluded individuals admitted in the ICU due to COVID-19.

Data preparation, quality control, analysis, and figure creation were carried out with the R software v.4.3.2 [24], except for the Flowchart that was created with Inkscape.

## 3 Results

**Fig. 1:**
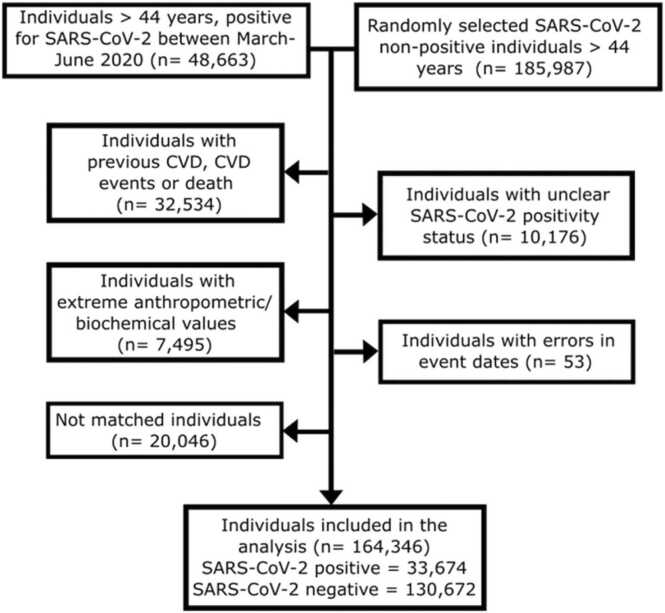
Flowchart of the included individuals. CVD: cardiovascular disease, SARS-CoV-2: severe acute respiratory syndrome coronavirus 2.

We included 164,346 individuals aged 45 years or older from the general population of Catalonia, northeastern Spain. Among them, 33,674 (20.5 %) tested positive for SARS-CoV-2 (Fig. 1). The mean follow-up duration was 317 days for CVD outcomes and 371 days for mortality.

### 3.1 Descriptive analysis

Comparison between matched and non-matched groups revealed that non-matched individuals were more frequently males (Table S5 in SI). Anthropometric, biochemical, and blood pressure data were similar in both groups, except for a higher concentration of triglycerides in non-matched individuals. Conversely, the prevalence of smoking, CVD risk factors, and other risk factors was lower in non-matched individuals.

Sociodemographic variables and risk factors were comparable between SARS-CoV-2 positive and non-positive individuals (Table 2). The mean age of the included individuals was 66 years, with approximately 60 % being females. While statistical differences were noted for some variables, the CIs of continuous variables were either overlapping or closely aligned. Additionally, percentages for most categorical variables did not substantially differ between groups, except for a higher prevalence of pre-existing renal failure and dementia among SARS-CoV-2 positive individuals.

**Table 2:** Baseline characteristics and previous clinical history of the matched population by positivity for SARS-CoV-2.

	SARS-CoV-2 non-positive n = 130,672	SARS-CoV-2 positive n = 33,674	p-value
***Sociodemographic variables***			
Age, years	66.0 (66.0, 66.0)	66.0 (66.0, 66.0)	0.070
Female sex, %	78,068 (59.7 %)	19,972 (59.3 %)	0.150
***Cardiovascular risk factors***			
Body mass index, kg/m^2^	27.4 (27.4, 27.5)	27.9 (27.8, 28.0)	< 0.001
Creatinine, mg/dL	0.82 (0.82, 0.82)	0.82 (0.82-0.83)	0.520
Renal failure, %	4524 (3.46 %)	2072 (6.15 %)	< 0.001
HDL cholesterol, mg/dL	55.3 (55.1, 55.5)	53.0 (52.8, 53.0)	< 0.001
LDL cholesterol, mg/dL	122 (122, 122)	120 (120, 121)	< 0.001
Total cholesterol, mg/dL	202 (202, 203)	197 (196, 198)	< 0.001
Triglycerides, mg/dL	111 (110, 111)	112 (111, 113)	0.001
Hypercholesterolemia, %	52,548 (56.0 %)	13,438 (52.3 %)	< 0.001
Cholesterol treatment* , %	28,164 (53.6 %)	7027 (52.3 %)	0.007
Glucose, mg/dL	95.0 (95.0, 95.0)	93.8 (93.6, 94.0)	< 0.001
Diabetes, %	21,781 (24.0 %)	6098 (24.2 %)	0.454
Diabetes treatment* , %	14,478 (66.5 %)	4010 (65.8 %)	0.306
Diastolic blood pressure, mmHg	76.0 (76.0, 76.0)	75.0 (75.0, 76.0)	< 0.001
Systolic blood pressure, mmHg	131 (131, 131)	130 (130, 130)	< 0.001
Hypertension, %	64,924 (69.5 %)	17,166 (69.2 %)	0.381
Hypertension treatment* , %	47,689 (73.5 %)	12,465 (72.6 %)	0.028
Smokers, %	13,973 (10.7 %)	3597 (10.7 %)	0.960
***Other risk factors***			
Cancer, %	8825 (6.75 %)	2404 (7.14 %)	0.013
COPD, %	2844 (2.18 %)	950 (2.82 %)	< 0.001
Dementia, %	1008 (0.77 %)	1727 (5.13 %)	< 0.001
Liver failure, %	68 (0.05 %)	28 (0.08 %)	0.048

Data are presented as median (95 % confidence interval) for continuous variables, and as number (percentage) for categorical variables. Continuous variables were compared between groups with the U-Mann-Whitney test, and categorical variables with the Chi-squared/Fisher exact tests. *Proportion treated was calculated as treated/individuals with the condition. COPD: chronic obstructive pulmonary disease; HDL: high-density lipoprotein; LDL: low-density lipoprotein; SARS-CoV-2: severe acute respiratory syndrome coronavirus 2.

Forty-five percent of SARS-CoV-2 positive individuals were hospitalized due to COVID-19, with an average hospitalization of 14 days duration. Among those hospitalized, 12 % were admitted to the ICU, 10 % received extracorporeal oxygenation or mechanical ventilation, and 9 % required tracheal drainage or intubation.

### 3.2 Outcome incidence in the follow-up

**Fig. 2:**
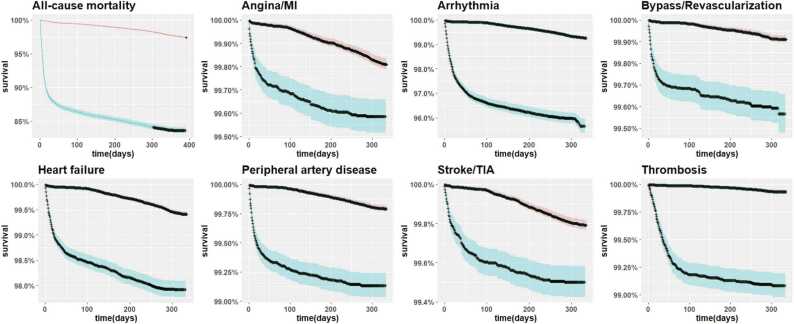
Kaplan-Meier survival curves with 95 % confidence intervals for each outcome during the 12-month follow-up in SARS-CoV-2 positive and non-positive individuals. The upper/pink curve represents the SARS-CoV-2 non-positive individuals, while the lower/blue curve represents the SARS-CoV-2 positive individuals. Arrhythmias include atrial fibrillation, flutter and tachycardia. MI: myocardial infarction, TIA: transient ischemic attack.

The crude incidence of all CVD outcomes and mortality throughout the follow-up was higher in SARS-CoV-2 positive versus non-positive individuals (Table 1). Incidence of thrombosis, mortality, and arrhythmias was highest among the SARS-CoV-2 positive individuals, with incidences approximately 11, 6, and 4 times higher, respectively, compared to their non-positive counterparts. The observed increase in CVD outcomes and mortality in SARS-CoV-2 positive individuals was particularly pronounced during the first 3 months of follow-up (Fig. 2). Crude regression estimates for the main analysis with matching are depicted in Table S6 from SI.

**Fig. 3:**
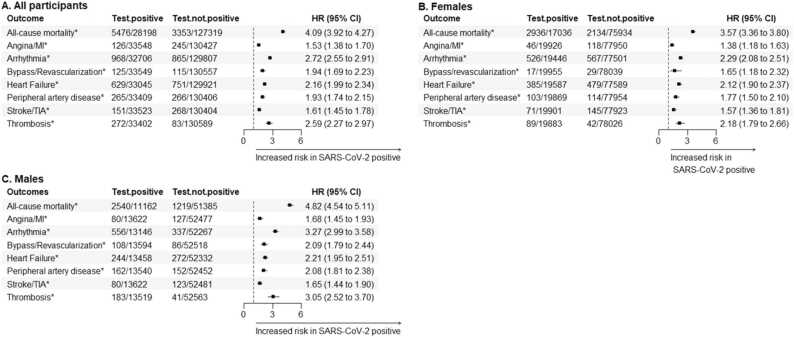
Hazard ratios (HR) and 95 % confidence intervals (CI) for the adjusted risk of cardiovascular outcomes and mortality during the 12-month follow-up in individuals positive for SARS-CoV-2, compared to non-positive individuals. Graphs show the estimates for all individuals (A), for females (B), and for males (C). Estimates were obtained with accelerated failure time models adjusted by age, blood pressure (diastolic and systolic), body mass index, cholesterol (HDL and total), and previous history of cancer, COPD, dementia, hypercholesterolemia, liver and renal insufficiency. Arrhythmias include atrial fibrillation, flutter and tachycardia. MI: myocardial infarction; TIA: transient ischemic attack. Outcomes showing a significant HR are highlighted with * .

**Fig. 4:**
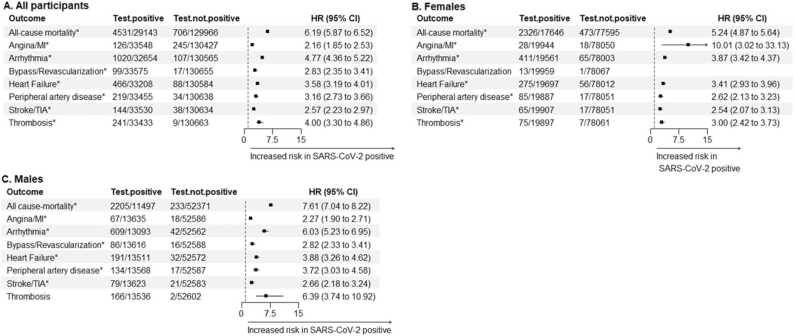
Hazard ratios (HR) and 95 % confidence intervals (CI) for the adjusted risk of cardiovascular outcomes and mortality during the first 3 months in individuals positive for SARS-CoV-2 compared to non-positive individuals. Graphs show the estimates for all individuals (A), for females (B), and for males (C). Estimates were obtained with accelerated failure time models adjusted by age, blood pressure (diastolic and systolic), body mass index, cholesterol (HDL and total), and previous history of cancer, COPD, dementia, hypercholesterolemia, liver and renal insufficiency. The model for bypass/revascularization in females could not be fitted. Arrhythmias include atrial fibrillation, flutter and tachycardia. MI: myocardial infarction; TIA: transient ischemic attack. Outcomes showing a significant HR are highlighted with * .

Adjusted models demonstrated that testing positive for SARS-CoV-2 was associated with a shorter time to all CVD events and mortality in the total sample, both at 3 months and 12 months of follow-up (Figs. 3A and 4A, respectively). The same results were observed when. analysis was stratified by sex (Figs. 3B, 3C, 4B, and 4C). Arrhythmias were the CVD event with the highest risk during the 12-month follow-up, with HR of 2.72 (2.55, 2.91) in both sexes, 2.29 (2.08, 2.51) in females, and 3.27 (2.99, 3.58) in males. During the first 3 months, the CVD events showing the highest risk were arrhythmias in the total sample (HR = 4.77 [4.36, 5.22]), coronary events in females (HR = 10.01 [3.02, 33.13]), and thrombosis in males (HR= 6.39 [3.74, 10.92]).

**Fig. 5:**
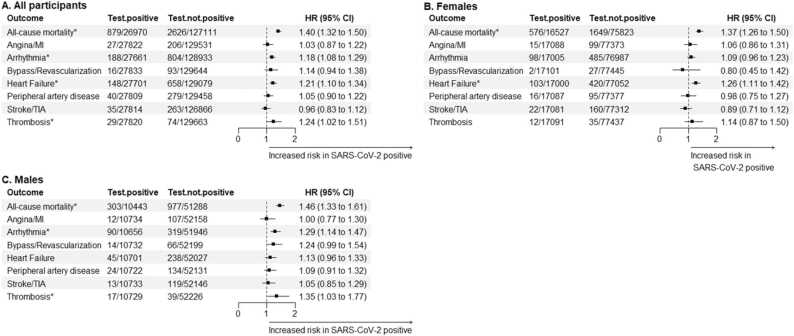
Hazard ratios (HR) and 95 % confidence intervals (CI) for the adjusted risk of cardiovascular outcomes and mortality from the 4th month to the end of the follow-up in individuals positive for SARS-CoV-2 compared to non-positive individuals. Graphs show the estimates for all individuals (A), for females (B), and for males (C). Estimates were obtained with accelerated failure time models adjusted by age, blood pressure (diastolic and systolic), body mass index, cholesterol (HDL and total), and previous history of cancer, COPD, dementia, hypercholesterolemia, liver and renal insufficiency. Arrhythmias include atrial fibrillation, flutter and tachycardia. MI: myocardial infarction; TIA: transient ischemic attack. Outcomes showing a significant HR are highlighted with * .

From the 4th month to the end of the follow-up period, adjusted analysis showed an association between SARS-CoV-2 positivity and mortality among all individuals, as well as in analysis stratified by sex (Fig. 5). Moreover, incidence of arrhythmias, heart failure, and thrombosis was increased in SARS-CoV-2 positive individuals during this period. Females exhibited an increased risk of heart failure (HR = 1.26 [1.11, 1.42], while males showed elevated risk of arrhythmias and thrombosis (HR = 1.29 [1.14, 1.47], and HR = 1.35 [1.03, 1.77], respectively).

### 3.3 Sensitivity analysis

The sensitivity analysis incorporating all individuals without matching produced similar findings (Tables S7-9 in SI). The sensitivity analysis excluding SARS-CoV-2 positive individuals admitted to the ICU also yielded comparable results (Tables S10-12 in SI). However, when ICU patients were excluded, incidence of thrombosis in SARS-CoV-2 positive males was no longer significantly increased during the 4- to 12-month follow-up period (Table S12 in SI).

## 4 Discussion

Our results showed a higher risk of all CVD events analyzed and of mortality among SARS-CoV-2 positive versus non-positive individuals, both within the initial 3-month period and throughout the 12-month follow-up. During the entire follow-up, arrhythmias were the CVD event showing the highest risk in the SARS-CoV-2 positive group. During the first 3 months, the highest risk was observed for coronary events in females and for thrombosis in males. We also observed an increased risk of bypass/revascularization, heart failure, peripheral artery disease, and stroke/TIA among SARS-CoV-2 positive individuals. In the second period of the follow-up (months 4–12), females positive for SARS-CoV-2 had a higher risk of heart failure, whereas males positive for SARS-CoV-2 showed an elevated risk of arrhythmias as well as thrombosis. Moreover, SARS-CoV-2 positive individuals faced an increased mortality risk. When individuals admitted to the ICU were excluded from the analysis, the risk of thrombosis in months 4–12 of follow-up was comparable between SARS-CoV-2 positive and non-positive men.

Previous studies have reported an increased incidence of CVD events within the first 24 months after SARS-CoV-2 positivity or COVID-19 infection [12], [13], [14], [15], [16], [17], [18], [19]. The association between CVD events and SARS-CoV-2 infection may be mediated by systemic inflammation induced by SARS-CoV-2, endothelial activation, and/or microvascular thrombosis, which could accelerate subclinical cardiovascular disease or cause new cardiovascular damage [1].

In line with our findings at 3 months and 12 months of follow-up, prior studies with longer follow-up indicate that SARS-CoV-2 positive individuals and COVID-19 patients have an increased risk of arrhythmias [12], [13], [15], [16], [19], heart failure [12], [13], [16], [19], ischemic heart disease [12], [13], [15], [17], [19], stroke/transient ischemic attack [12], [13], [15], [18], [19] and thrombosis [12], [13], [15], [19]. Furthermore, some studies have reported an increased risk of cardiac arrest [12], [13], cardiogenic shock [12], [13], cardiomyopathy [12], [13], [16], and inflammatory heart disease [12], [13], [15]. Interestingly, our study identified an increased risk of bypass/revascularization and of peripheral artery disease in SARS-CoV-2 positive individuals, which had not been reported previously. This elevated risk of bypass/revascularization is probably a consequence of the higher incidence of ischemic heart disease in SARS-CoV-2 positive individuals. Other authors suggest that the increased occurrence of peripheral artery disease could be due to worsening arterial stiffness following SARS-CoV-2 infection [25], [26].

We observed that the CVD events with the highest risk in SARS-CoV-2 positive individuals were arrhythmias, coronary events, and thrombosis during the first 3 months and arrhythmias during the entire 12-month follow-up. In previous studies in SARS-CoV-2 positive individuals and in COVID-19 patients, the highest risk CVD events were arrhythmias [16], heart failure [19], thrombosis [12], [15], and inflammatory heart disease [13]. It is plausible that arrhythmias show high incidence soon after SARS-CoV-2 infection because they are associated with inflammation, myocardial ischemia, and pre-existing heart conditions [27]. On the other hand, the incidence of thrombosis may be triggered by endothelial activation, hypercoagulation, inflammation, and platelet activation induced by SARS-CoV-2 infection [28].

From the fourth month onward, we found an increased risk of arrhythmias, heart failure, and thrombosis in individuals positive for SARS-CoV-2; however, the other CVD events analyzed showed comparable incidence in both positive and non-positive individuals. Many studies have reported an increased risk of most CVD events within the first 2 years after SARS-CoV-2 and COVID-19 positivity [12], [19]; the absence of studies excluding early CVD events (e.g., up to 3 months post-infection with SARS-CoV-2) may contribute to this seemingly dire finding at 2 years of follow-up [12], [19]. Interestingly, a study focusing on patients hospitalized with COVID-19 identified heart failure as the most common CVD-related rehospitalization in these patients [29].

Our results showed that during the initial 3 months and throughout the entire 12-month follow-up, SARS-CoV-2 positive individuals exhibited a higher incidence of all CVD events compared to non-positive individuals, irrespective of sex. However, sex disparities emerged after excluding events in the initial 3 months. From the fourth month onward, we observed an elevated risk of heart failure among SARS-CoV-2 positive females, while SARS-CoV-2 positive males showed an increased risk of arrhythmias and of thrombosis. Three studies provide conflicting sex-stratified results on CVD incidence after SARS-CoV-2 infection, with approximately 1 year of follow-up [12], [13], [16]. One study reported a higher incidence of arrhythmias, cardiac arrest, cardiogenic shock, cardiomyopathy, cerebrovascular events, inflammatory heart disease, ischemic heart disease, and heart failure in SARS-CoV-2 positive individuals, showing similar results in females and males [13]. Another study found a similar CVD event incidence, except for cerebrovascular disorders, which showed a higher incidence only in males [12]. The third study identified an increased risk of arrhythmias in SARS-CoV-2 positive individuals, a higher risk of angina and heart failure in the subset of females, and a higher risk of cardiomyopathy in the subset of males [16]. These sex-stratified discrepancies in CVD event incidence after SARS-CoV-2 infection could be related to the sample size of these studies and to inherent CVD differences between females and males. In the general population, most heart failure patients are females and the population-attributable rate of heart failure risk factors is quite distinct between females and males [30]. In addition, the incidence of arrhythmia subtypes varies by sex; this variation could be caused by hormonal effects on ion channels and/or by dissimilar autonomic tone, among other factors [31].

When COVID-19 patients admitted to the ICU were excluded from analysis, the previously observed increase of thrombosis during the last period of the follow-up (4–12 months) in SARS-CoV-2 positive males became nonsignificant. This finding is coherent with prior research showing that the elevated risk of thrombosis in SARS-CoV-2 positive individuals was only significant in hospitalized patients [16].

In our study, the mortality risk was higher in SARS-CoV-2 positive individuals, both within the initial 3 months post-infection and beyond. This result aligns with existing literature demonstrating an elevated risk of all-cause mortality after SARS-CoV-2 positivity and after COVID-19 in both the short and the long term [13], [14], [17], [19]. The long-term mortality risk after SARS-CoV-2 infection could be caused by CVD events, cancer progression [33], or pulmonary complications such as fibrosis and hypertension [32]. Disparities related to race/ethnicity, healthcare access, and socioeconomic status may further exacerbate mortality risk after COVID-19 [34].

The main strengths of our study are its large sample size, the rigorous definition of SARS-CoV-2 non-positive individuals, and the robust statistical methods used. We included 164,346 individuals with data on the electronic health records from a region of Spain with nearly 8 million inhabitants. Our strict definition of non-positive SARS-CoV-2 individuals required the exclusion of records flagged as suspicious in COVID-19 epidemiological registries. Furthermore, we employed consistent statistical methods such as matching, robust standard errors, and sensitivity analyses.

On the other hand, our study has some limitations that should be acknowledged. First, statistical power constraints prevented the analysis of certain individual outcomes. However, we examined 7 CVD outcomes covering a broad spectrum of CVD events associated with SARS-CoV-2, including 3 individual outcomes. Second, our study population may not precisely represent the general population. Smoking prevalence was lower in the included cohort than in the general population, which may reflect underreporting of smoking status in primary care and in hospital admissions. On the other hand, hypercholesterolemia and hypertension prevalence were higher in this sample than in the general population. This observation could be due to lack of awareness of these conditions in the general population and/or to an excess of comorbidities in selected individuals, compared to the general population. At the same time, a reference group with more comorbidities would reduce the observed differences between groups. Third, the observed effect of SARS-CoV-2 positivity on CVD incidence could differ for individuals infected in later periods of the COVID-19 pandemic, due to the distinct SARS-CoV-2 variants that were predominant in each period. The prevalence of cardiovascular symptoms was higher in individuals infected with the pre-Delta and Delta variants, compared to those infected with the omicron variant [35]. However, differences in cardiovascular symptom prevalence were not significant when the analysis was adjusted for vaccination. Therefore, we would expect a similar incidence of CVD in vaccinated individuals regardless of the SARS-CoV-2 variant they were infected with. Fourth, we could not examine the effect of SARS-CoV-2 vaccination and/or antibody titer in the association between SARS-CoV-2 positivity and cardiovascular outcomes/mortality, as these data were not available. It has been shown that the stronger the antibody response against SARS-CoV-2, the lower the mortality of infected individuals [36], [37]. Thus, we would expect that CVD incidence and mortality would be lower in vaccinated individuals or in those with a high anti-SARS-CoV-2 antibody titer, compared to non-vaccinated individuals or those with a low anti-SARS-CoV-2 antibody titer. Finally, our risk estimates for the SARS-CoV-2 positive group could be larger than the population estimates due to the characteristics of the non-positive group. However, a large study of long-term CVD events showed that COVID-19 patients had an increased risk of all CVD events examined, irrespective of the reference group used [15].

## 5 Conclusions

In addition to previously reported CVD outcomes in SARS-CoV-2 and COVID-19, our findings showed an increased risk of bypass/revascularization and of peripheral artery disease in SARS-CoV-2 positive individuals within the first 3 months post-infection, compared to non-positive individuals. Our results from a 12-month follow-up also suggest that the major differences in CVD outcomes between SARS-CoV-2 positive and non-positive individuals manifest during the first 3 months post-infection and stabilize thereafter. However, the risk of arrhythmias, heart failure, and thrombosis differed by sex and persisted beyond the first 3 months of follow-up.

## Authors’ contributions

The authors accept full responsibility for the manuscript content. IRD conceptualized the study, acquired funding, administered the project and the resources, supervised the study, and created the original draft of the manuscript. RE and JM collaborated in conceptualization of the study, funding acquisition and resources. CT, JC, and IRD did the formal data analysis, validation, and visualization. ACV and IS did the data curation. All authors participated in the investigation and methodology, in reviewing and editing the original draft manuscript, and all approved the submitted manuscript.

## Declaration of Competing Interest

None.

## Data Availability

The data that support the findings of this study are available from the corresponding author.
