# Supplementary material for: Increased risk of arrhythmias, heart failure, and thrombosis in SARS-CoV-2 positive individuals persists at one year post-infection
Source: Comput Struct Biotechnol J. 2024 Jun 20;24:476–83. doi: 10.1016/j.csbj.2024.06.024 (PMC11266869; doi:10.1016/j.csbj.2024.06.024)
Supplement: Figure S1 — Supplementary material [file mmc1.docx]

**Supplementary information**

**Supplementary Figure S1.** Study timeline.

**Supplementary Figure S2.** Age balance in SARS-CoV-2 positive and non-positive individuals in 5 strata according to the propensity score.

**Supplementary Figure S3.** Sex balance in SARS-CoV-2 positive and non-positive individuals in 5 strata according to the propensity score.

**Supplementary Figure S4.** Balance for smoking habit in SARS-CoV-2 positive and non-positive individuals in 5 strata according to the propensity score.

**Supplementary Figure S5.** Balance for previous diabetes in SARS-CoV-2 positive and non-positive individuals in 5 strata according to the propensity score.

**Supplementary Figure S6.** Balance for previous hypercholesterolemia in SARS-CoV-2 positive and non-positive individuals in 5 strata according to the propensity score.

**Supplementary Figure S7.** Balance for previous hypertension in SARS-CoV-2 positive and non-positive individuals in 5 strata according to the propensity score.

**Supplementary Figure S8.** Balance for previous liver failure in SARS-CoV-2 positive and non-positive individuals in 5 strata according to the propensity score.

**Supplementary Figure S9.** Balance for previous renal failure in SARS-CoV-2 positive and non-positive individuals in 5 strata according to the propensity score.

**Supplementary Figure S10.** Balance for previous cancer in SARS-CoV-2 positive and non-positive individuals in 5 strata according to the propensity score.

**Supplementary Figure S11.** Balance for previous chronic obstructive pulmonary disease in SARS-CoV-2 positive and non-positive individuals in 5 strata according to the propensity score.

**Supplementary Figure S12.** Log(hazard ratios) and 95% confidence intervals for the adjusted risk of cardiovascular outcomes and mortality during the whole follow-up in individuals positive for SARS-CoV-2 compared to non-positive individuals.

**Supplementary Figure S13.** Log(hazard ratios) and 95% confidence intervals for the adjusted risk of cardiovascular outcomes and mortality during the first 3 months in individuals positive for SARS-CoV-2 compared to non-positive individuals.

**Supplementary Figure S14.** Log(hazard ratios) and 95% confidence intervals for the adjusted risk of cardiovascular outcomes and mortality from the 4^th^ month to the end of the follow-up in individuals positive for SARS-CoV-2 compared to non-positive individuals.

**Supplementary Table S1.** Included values for continuous variables.

**Supplementary Table S2.** International Classification of Diseases (ICD) codes used to obtain data on previous clinical history, cardiovascular risk factors and cardiovascular events.

**Supplementary Table S3.** Anatomical Therapeutic Chemical (ATC) codes used to obtain data on diabetes, hypercholesterolemia and hypertension treatment.

**Supplementary Table S4.** Balance in the covariates included in the propensity score model to match participants positive and non-positive for SARS-CoV-2.

**Supplementary Table S5.**  Baseline characteristics and previous clinical history of the study population by matching status.

**Supplementary Table S6.** Crude estimates for the main analysis with matching obtained with accelerated failure time models.

**Supplementary Table S7.** Estimates for the whole period in the sensitivity analysis without matching obtained with accelerated failure time models.

**Supplementary Table S8.** Estimates for the first 3 months in the sensitivity analysis without matching obtained with accelerated failure time models.

**Supplementary Table S9.** Estimates for the period 4 months – end of the follow-up in the sensitivity analysis without matching obtained with accelerated failure time models.

**Supplementary Table S10.** Estimates for the whole period in the sensitivity analysis excluding SARS-CoV-2 positive individuals admitted in the ICU for COVID-19 obtained with accelerated failure time models.

**Supplementary Table S11.** Estimates for the first 3 months in the sensitivity analysis excluding SARS-CoV-2 positive individuals admitted in the ICU for COVID-19 obtained with accelerated failure time models.

**Supplementary Table S12.** Estimates for the period 4 months – end of the follow-up in the sensitivity analysis excluding SARS-CoV-2 positive individuals admitted in the ICU for COVID-19 obtained with accelerated failure time models.

**Supplementary Figure S1.** Study timeline. CVD: cardiovascular disease

**
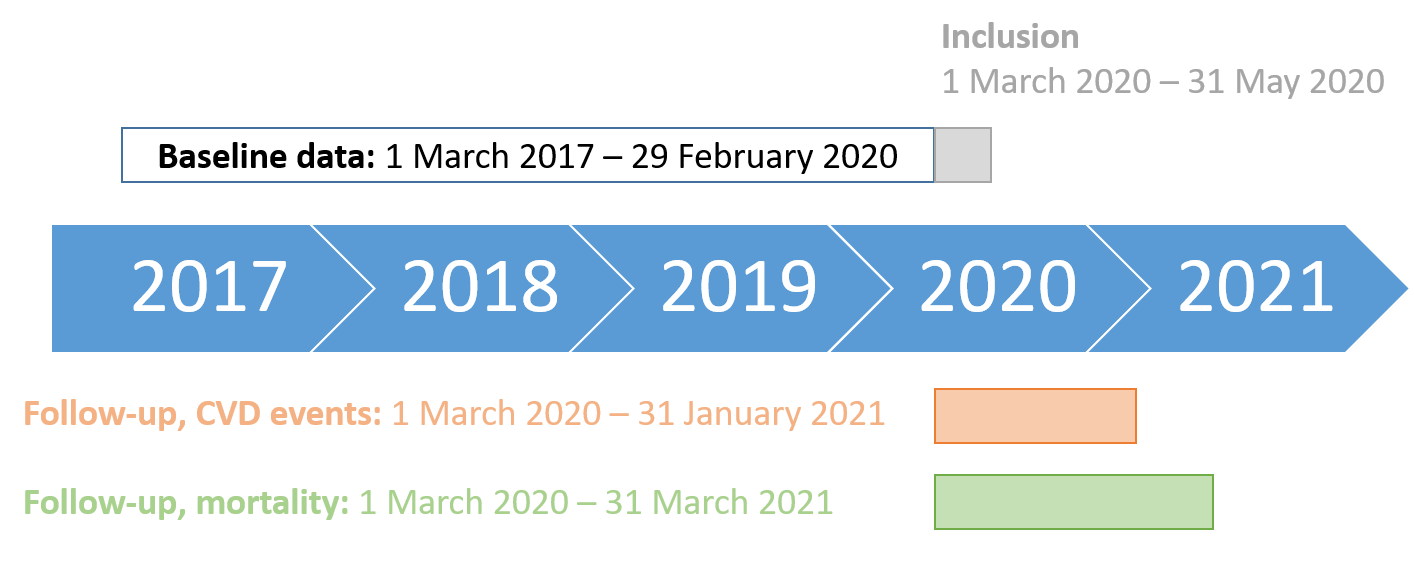
**

**Supplementary Figure S2.** Age balance in SARS-CoV-2 positive and non-positive individuals in 5 strata according to the propensity score. For each stratum, yellow boxplots represent SARS-CoV-2 non-positive individuals and orange boxplot represent SARS-CoV-2 positive individuals. Age comparison in each stratum is shown with a black line. The slope of the black line represents the mean age difference of age between groups.

**
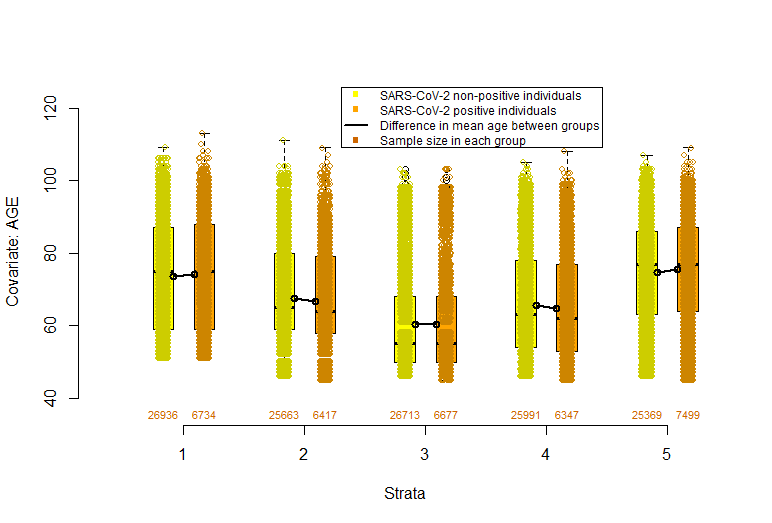
**

**Supplementary Figure S3.** Sex balance in SARS-CoV-2 positive and non-positive individuals in 5 strata according to the propensity score.


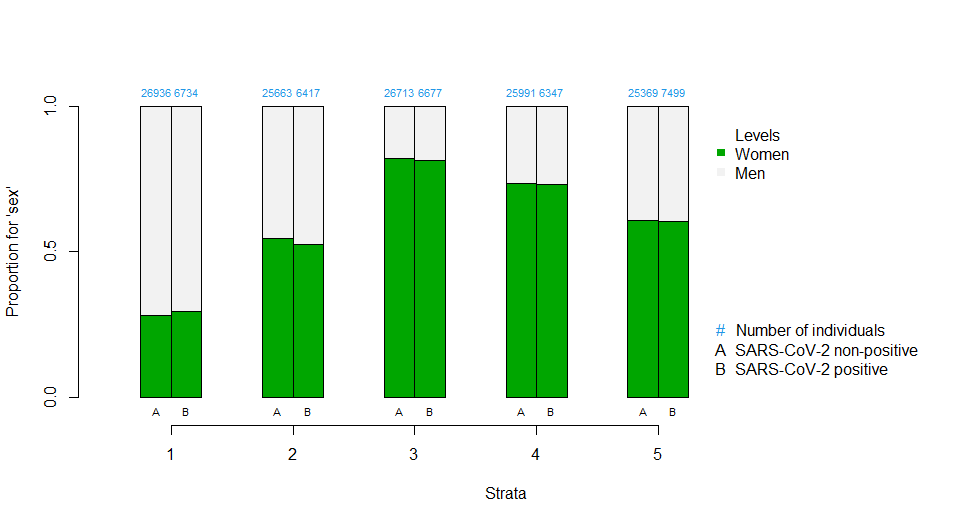


**Supplementary Figure S4.** Balance for smoking in SARS-CoV-2 positive and non-positive individuals in 5 strata according to the propensity score.

**
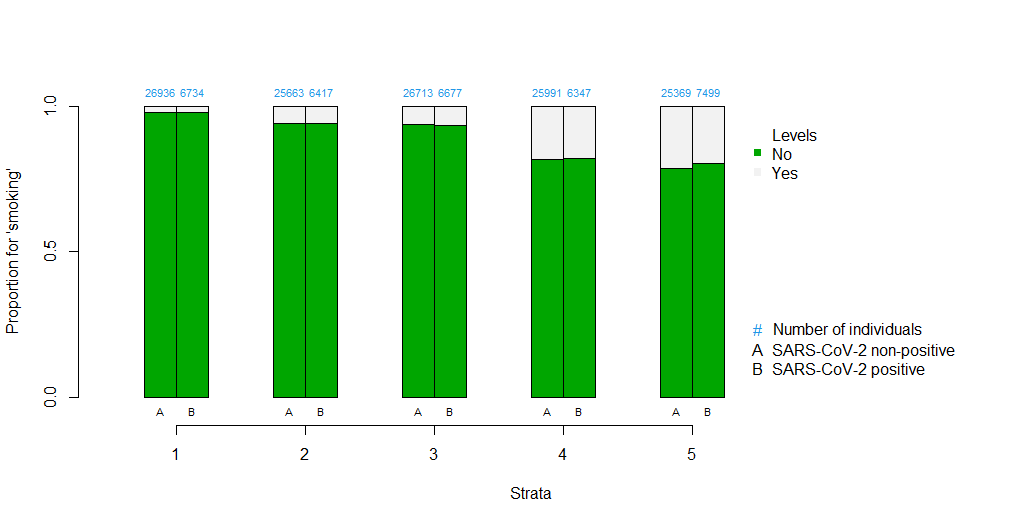
**

**Supplementary Figure S5.** Balance for previous diabetes in SARS-CoV-2 positive and non-positive individuals in 5 strata according to the propensity score.


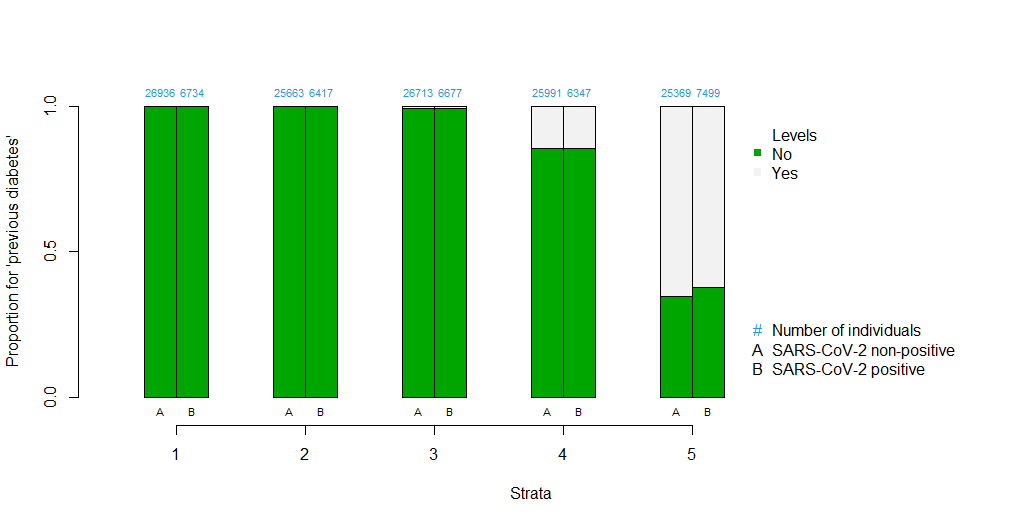


**Supplementary Figure S6.** Balance for previous hypercholesterolemia in SARS-CoV-2 positive and non-positive individuals in 5 strata according to the propensity score.


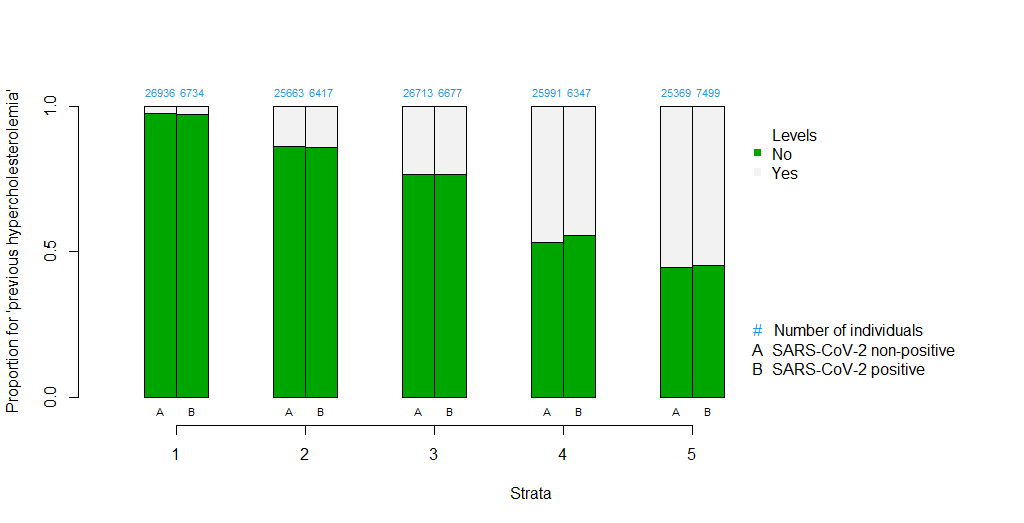


**Supplementary Figure S7.** Balance for previous hypertension in SARS-CoV-2 positive and non-positive individuals in 5 strata according to the propensity score.


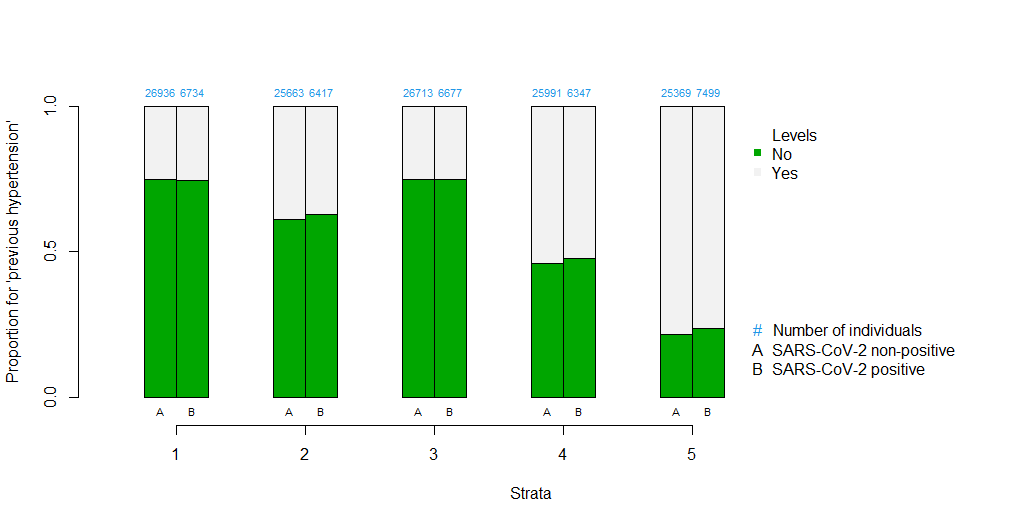


**Supplementary Figure S8.** Balance for previous liver failure in SARS-CoV-2 positive and non-positive individuals in 5 strata according to the propensity score.

**
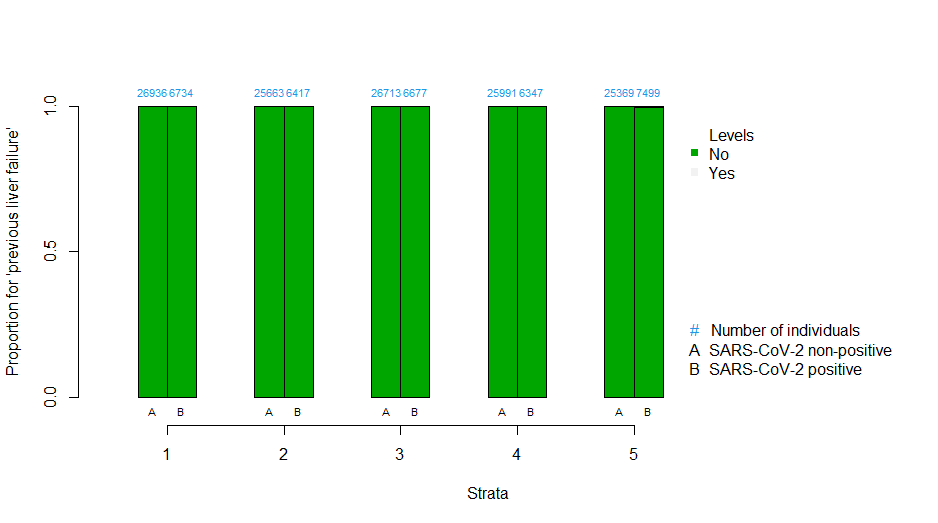
**

**Supplementary Figure S9.** Balance for previous renal failure in SARS-CoV-2 positive and non-positive individuals in 5 strata according to the propensity score.


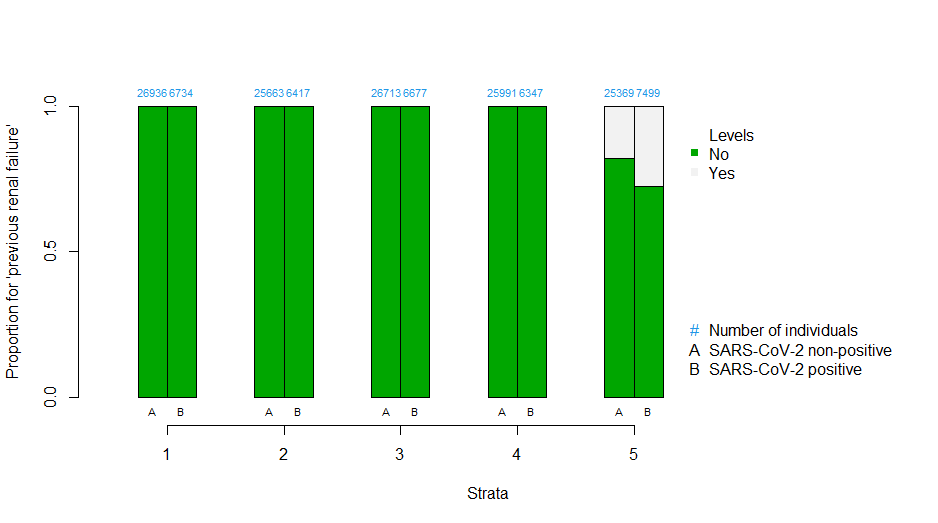


**Supplementary Figure S10.** Balance for previous cancer in SARS-CoV-2 positive and non-positive individuals in 5 strata according to the propensity score.


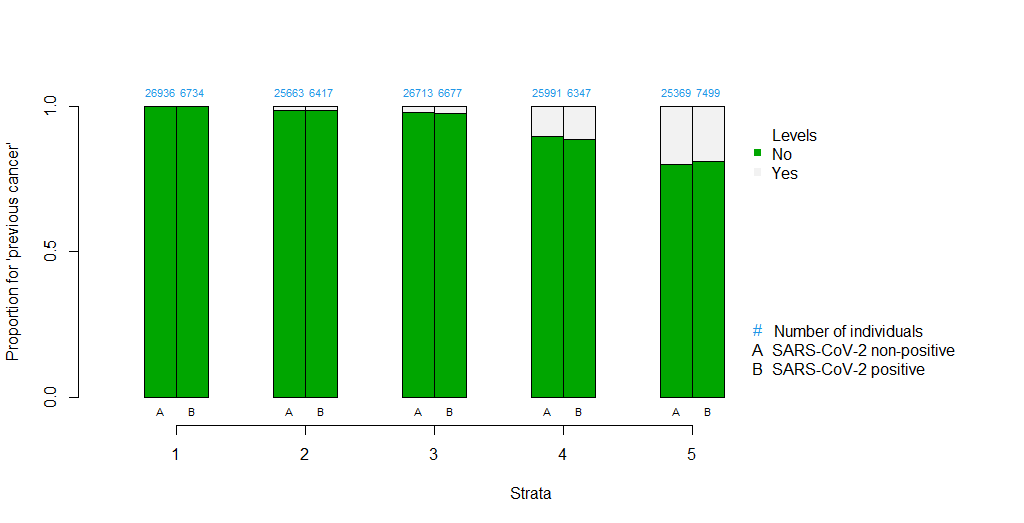


**Supplementary Figure S11.** Balance for previous chronic obstructive pulmonary disease in SARS-CoV-2 positive and non-positive individuals in 5 strata according to the propensity score.


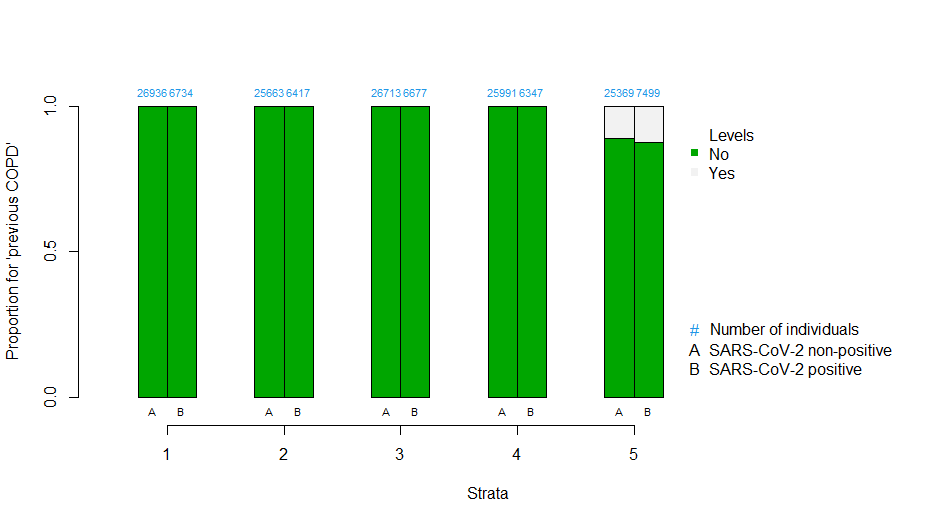


**Supplementary Figure S12.** Log(hazard ratios) and 95% confidence intervals for the adjusted risk of cardiovascular outcomes and mortality during the whole follow-up in individuals positive for SARS-CoV-2 compared to non-positive individuals. Estimates were obtained with accelerated time failure models. CI: confidence interval; HR: hazard ratio; MI: myocardial infarction; TIA: transient ischemic attack.


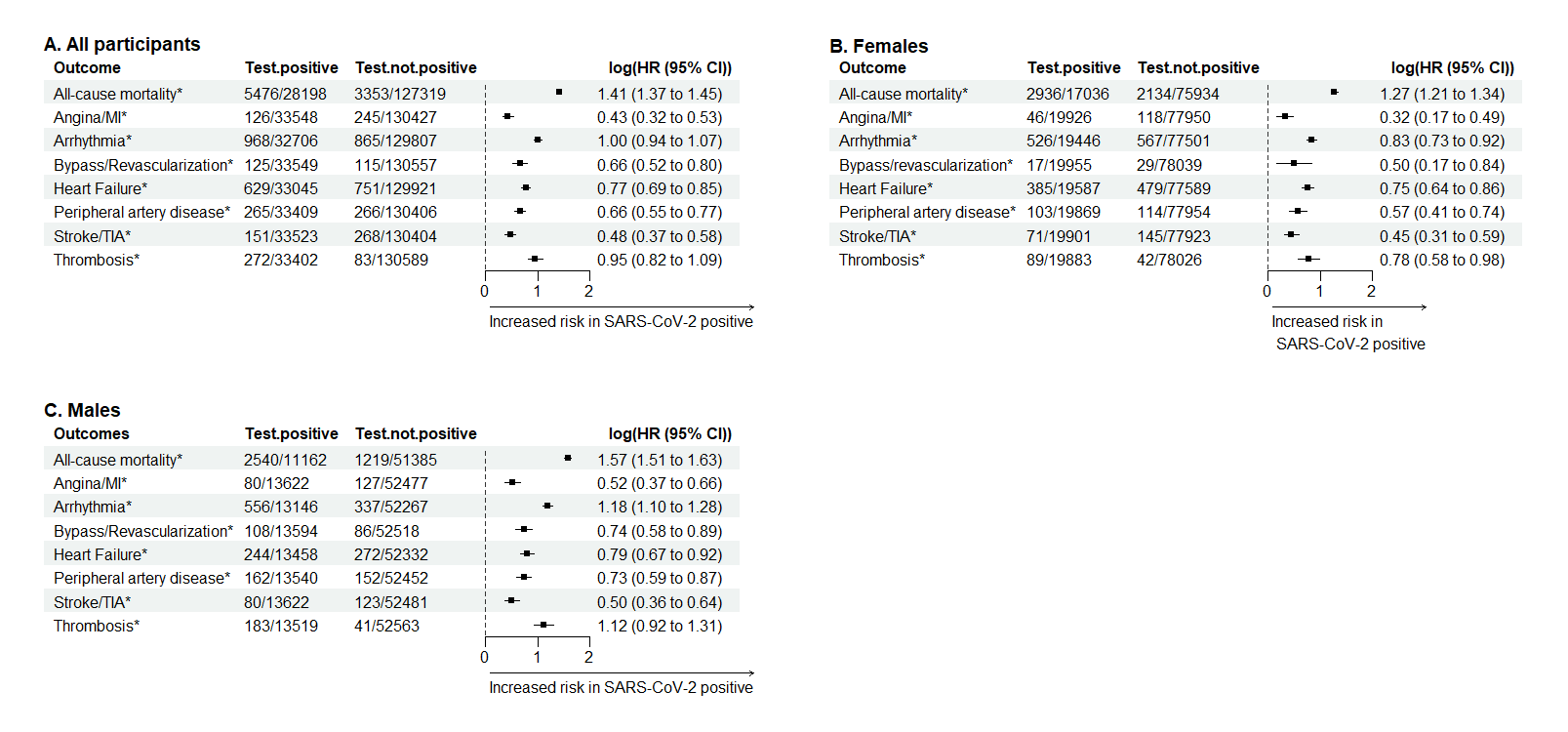


**Supplementary Figure S13.** Log(hazard ratios) and 95% confidence intervals for the adjusted risk of cardiovascular outcomes and mortality during the first 3 months in individuals positive for SARS-CoV-2 compared to non-positive individuals. Estimates were obtained with accelerated time failure models. CI: confidence interval; HR: hazard ratio; MI: myocardial infarction; TIA: transient ischemic attack.


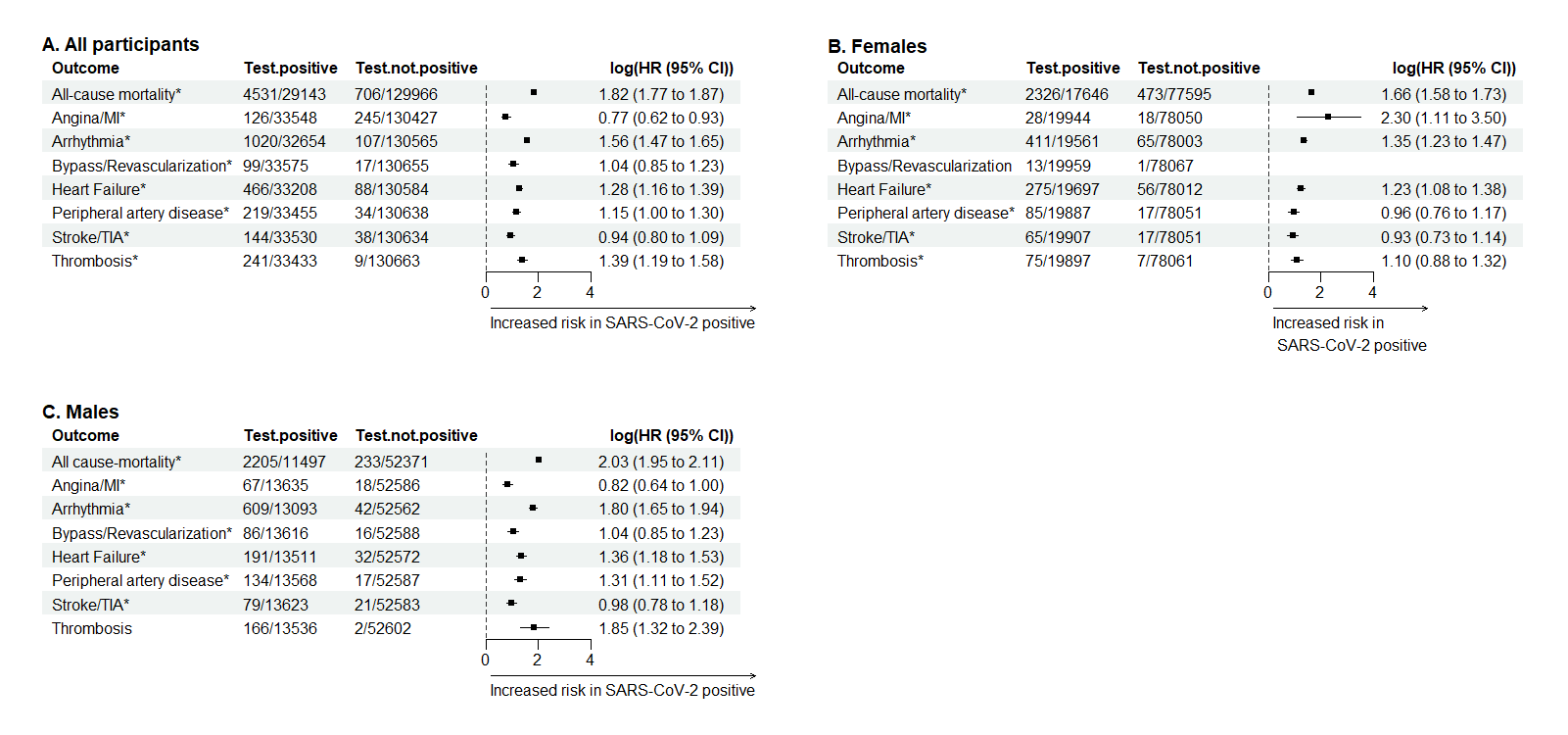


**Supplementary Figure S14.** Log(hazard ratios) and 95% confidence intervals for the adjusted risk of cardiovascular outcomes and mortality from the 4^th^ month to the end of the follow-up in individuals positive for SARS-CoV-2 compared to non-positive individuals. Estimates were obtained with accelerated time failure models. CI: confidence interval; HR: hazard ratio; MI: myocardial infarction; TIA: transient ischemic attack.


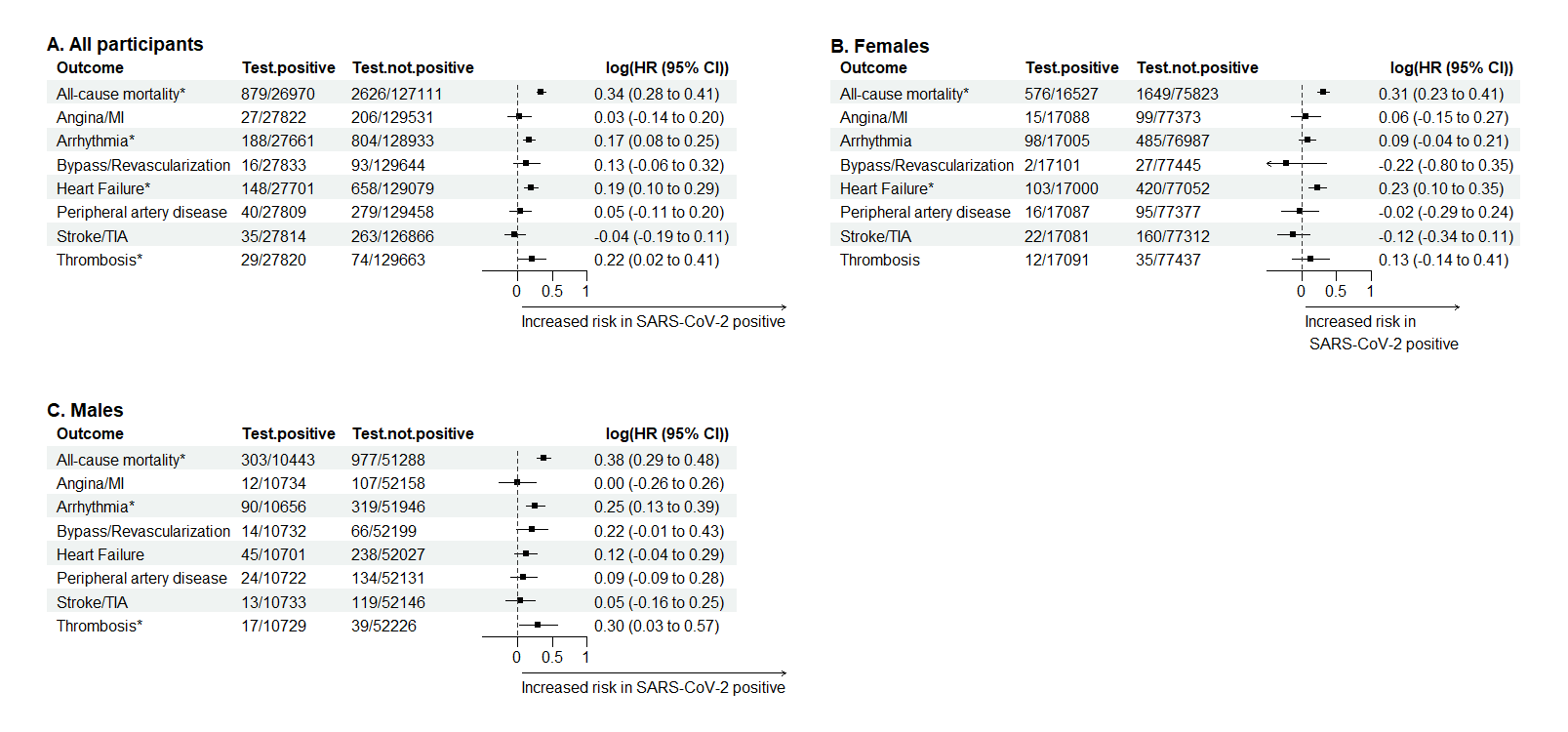


**Supplementary Table S1.** Included values for continuous variables

|  | **Minimum** | **Maximum** |
| --- | --- | --- |
| **Diastolic blood pressure** | 40 | 130 |
| **Systolic blood pressure** | 60 | 220 |
| **Body mass index** | 15 | 40 |
| **HDL cholesterol** | 20 | 130 |
| **LDL cholesterol** | 20 | 250 |
| **Total cholesterol** | 100 | 450 |
| **Creatinine** | 0.2 | 5.0 |
| **Glucose** | 30 | 350 |
| **Height** | 120 | 250 |
| **Triglycerides** | 20 | 400 |
| **Weight** | 35 | 150 |

HDL: high-density lipoprotein; LDL: low-density lipoprotein

**Supplementary Table S2.** International Classification of Diseases (ICD) codes used to obtain data on previous clinical history, cardiovascular risk factors and cardiovascular events.

|  | **ICD-9** | **ICD-10** |
| --- | --- | --- |
| ***Events and previous history*** |  |  |
| Angina | 413, 4130, 4131, 4132, 4133, 4138, 4139 | I20, I200, I201, I208, I209 |
| Atrial fibrillation and flutter | 42731, 42732 | I48 |
| Bypass | V4581 | Z951 |
| Cancer | 140-209 | C00-C97 |
| COPD | 49122, 49321, 49121, 49322, 49120, 49320, 496 | J44 |
| Dementia | 29040, 29041, 29410, 29411, 29420, 29421, 3310, 3308, 33182, 33183, 3316, 33189 | F00, F01, F028, F03, G30, G318, G310 |
| Heart failure | 40201, 40211, 40291, 40401, 40411, 40491, 40403, 40413, 40493, 428.XX | I50 |
| Liver failure | 570, 5722, 5728, 57141, 57149, 57140, 5715, 5719, 5716, 7824 | K72, K73, K74, R17 |
| Myocardial infarction | 410, 4100, 41000, 41001, 41002, 4101, 41010, 41011, 41012, 4102, 41020, 41021, 41022, 4103, 41030, 41031, 41032, 4104, 41040, 41041, 41042, 4105, 41050, 41051, 41052, 4106, 41060, 41061, 41062, 4107, 41070, 41071, 41072, 4108, 41080, 41081, 41082, 4109, 41090, 41091, 41092 | I21, I210, I211, I212, I213, I214, I219, I22, I220, I221, I228, I229, I23, I230, I231, I232, I233, I234, I235, I236, I238 |
| Peripheral artery disease | 44020, 44021, 44022, 44023, 44024, 44029, 4403, 44030, 44031, 44032, 4404, 4439 | I70, I702, I708, I709, I73, I738, I739 |
| Renal failure | 5845, 5836, 5846, 5837, 5847, 5848, 5849, 5851, 5852, 5853, 5854, 5855, 5856, 5859, 586 | N17, N18, N19 |
| Revascularization | V4582 | Z955 |
| Stroke | 433, 4330, 43301, 4331, 43311, 4332, 43321, 4333, 43331, 4338, 43381, 4339, 43391, 434, 4340, 43401, 4341, 4342, 4349, 43491, 438,4380, 4381, 43810, 43811, 43812, 43813, 43814, 43819, 4382, 43820, 43821, 43822, 4383, 43830, 43831, 43832, 4384, 43840, 43841, 43842, 4385, 43850, 43851, 43852, 43853, 4386, 4387, 4388, 43881, 43882, 43883, 43884, 43885, 43889, 4389 | I63, I630, I632, I633, I635, I636, I638, I639, I64, I65, I650, I651, I652, I653, I658, I659 |
| Tachycardia | 4270, 4271, 7850 | I471, I472, R000 |
| Thromboembolism | 44401, 44409, 4441, 44421, 44422, 44481, 44489, 4449, 4530, 4531, 45387, 45377, 4532, 4533, 45340, 45341, 45342, 45350, 45351, 45352, 45383, 45381, 45382, 45373, 45371, 45372, 45384, 45374, 45385, 45375, 45386, 45376, 4536, 45389, 45379 | I74, I82 |
| Transient ischemic attack | 435 | G45-G46 |
| ***Risk factors*** |  |  |
| Diabetes | 25011, 25031, 25041, 25051, 25061, 25071, 25081, 25091, 25001, 25020, 25010, 25030, 25040, 25050, 25060, 25070, 25080, 25090, 25000, 24920, 24910, 24930, 24940, 24950, 24960, 24970, 24980, 24990, 24900 | E10, E11, E12, E13, E14 |
| Dyslipidemia | 2720, 2722, 2724, 2728, 2729 | E780, E782, E784, E785, E788, E789 |
| Hypertension | 4010, 4011, 4019, 40201, 40211, 40291, 40200, 40210, 40290, 40301, 40311, 40391, 40300, 40310, 40390, 40401, 40411, 40491, 40400, 40410, 40490, 40402, 40412, 40492, 40403, 40413, 40493, 40501, 40511, 40591, 40599, 40509, 40519 | I10, I11, I12, I13, I15 |
| Smoking | 3051 | Z720 |

**Supplementary Table S3.** Anatomical Therapeutic Chemical (ATC) codes used to obtain data on diabetes, hypercholesterolemia and hypertension treatment.

|  | **ATC code** |
| --- | --- |
| **Angiotensin converting enzyme inhibitors** | C09A, C09B |
| **Angiotensin receptor blockers** | C09C, C09D |
| **Antihypertensive drugs** | C02 |
| **Beta blockers** | C07 |
| **Calcium channel blockers** | C08CA, C08D |
| **Diuretics** | C03 |
| **Drugs used in diabetes mellitus** | A10 |
| **Other lipid-lowering drugs** | C10AB, C10AC, C10AD, C10AX |
| **Statins** | C10AA, C10B |

**Supplementary Table S4.** Balance in the covariates included in the propensity score model to match participants positive and non-positive for SARS-CoV-2.

|  | **Adjusted standardized difference in means** |
| --- | --- |
| **Age** | -0.0122 |
| **Sex** | 0.0071 |
| **Previous cancer** | 0.0024 |
| **Previous COPD** | 0.0147 |
| **Previous diabetes** | 0.0112 |
| **Previous hypercholesterolemia** | -0.0100 |
| **Previous hypertension** | -0.0226 |
| **Previous liver insufficiency** | 0.0032 |
| **Previous renal insufficiency** | 0.0029 |
| **Previous smoking** | -0.0062 |

Balance is considered adequate when adjusted standardized differences in means are <0.1 for all variables.

**Supplementary Table S5.**  Baseline characteristics and previous clinical history of the study population by matching status.

|  | **Matched**  **n= 164,346** | **Non-matched**  **n= 20,046** | **p-value** |
| --- | --- | --- | --- |
| ***Sociodemographic variables*** |  |  |  |
| Age, years | 66.0 (66.0, 66.0) | 66.0 (66.0, 67.0) | <0.001 |
| Female sex, % | 98,040 (59.7%) | 9,896 (49.4%) | <0.001 |
| ***Cardiovascular risk factors*** |  |  |  |
| Body mass index, kg/m^2^ | 27.5 (27.5, 27.6) | 26.8 (26.6, 26.9) | <0.001 |
| Creatinine, mg/dL | 0.82 (0.82, 0.82) | 0.84 (0.83, 0.84) | 0.002 |
| Renal failure, % | 6,596 (4.01%) | 2 (0.01%) | <0.001 |
| HDL cholesterol, mg/dL | 54.9 (54.8, 55.0) | 57.0 (56.5, 57.1) | <0.001 |
| LDL cholesterol, mg/dL | 122 (122, 122) | 124 (123, 124) | <0.001 |
| Total cholesterol, mg/dL | 201 (201, 202) | 202 (201, 203) | 0.197 |
| Triglycerides, mg/dL | 111 (111, 112) | 99.2 (98.0, 100) | <0.001 |
| Hypercholesterolemia, % | 65,986 (55.2%) | 4,092 (38.1%) | <0.001 |
| Cholesterol treatment*, % | 35,191 (53.3%) | 2,191 (53.5%) | 0.804 |
| Glucose, mg/dL | 94.9 (94.7, 95.0) | 91.8 (91.5, 92.0) | <0.001 |
| Diabetes, % | 27,879 (24.0%) | 264 (2.49%) | <0.001 |
| Diabetes treatment*, % | 18,488 (66.3%) | 99 (37.5%) | <0.001 |
| Diastolic blood pressure, mmHg | 76.0 (76.0, 76.0) | 75.0 (75.0, 76.0) | 0.005 |
| Systolic blood pressure, mmHg | 131 (130, 131) | 130 (130, 130) | <0.001 |
| Hypertension, % | 82,090 (69.4%) | 5,718 (52.7%) | <0.001 |
| Hypertension treatment*, % | 60,154 (73.3%) | 3,989 (69.8%) | <0.001 |
| Smokers, % | 17,570 (10.7%) | 643 (3.21%) | <0.001 |
| ***Other risk factors*** |  |  |  |
| Cancer, % | 11,229 (6.83%) | 227 (1.31%) | <0.001 |
| COPD, % | 3,794 (2.31%) | 1 (0.00%) | <0.001 |
| Dementia, % | 2,735 (1.66%) | 76 (0.38%) | <0.001 |
| Liver failure, % | 96 (0.06%) | 2 (0.01%) | 0.008 |

Data is presented as median (95% confidence interval) for continuous variables, and as number (percentage) for categorical variables. Continuous variables were compared between groups with the U-Mann-Whitney test, and categorical variables with the chi-squared/exact tests. *Proportion of treated was calculated as treated/individuals with the condition. COPD: chronic obstructive pulmonary disease; HDL: high-density lipoprotein; LDL: low-density lipoprotein.

**Supplementary Table S6.** Crude estimates for the main analysis with matching obtained with accelerated failure time models.

|  | **All participants**  **HR (95% CI)** | **Females**  **HR (95% CI)** | **Males**  **HR (95% CI)** |
| --- | --- | --- | --- |
| **Whole follow-up** | | | |
| Angina / MI | 1.39 (1.29, 1.50)* | 1.24 (1.11, 1.38)* | 1.53 (1.40, 1.70)* |
| AF / flutter / tachycardia | 2.17 (2.08, 2.27)* | 1.86 (1.75, 1.97)* | 2.60 (2.44, 2.77)* |
| Bypass / revascularization | 1.69 (1.52, 1.88)* | 1.38 (1.10, 1.74)* | 1.84 (1.63, 2.08)* |
| All-cause mortality | 2.85 (2.78, 2.92)* | 2.60 (2.51, 2.68)* | 3.24 (3.13, 3.36)* |
| Heart failure | 1.71 (1.63, 1.80)* | 1.69 (1.59, 1.80)* | 1.75 (1.62, 1.89)* |
| Peripheral artery disease | 1.70 (1.58, 1.83)* | 1.62 (1.45, 1.81)* | 1.79 (1.62, 1.98)* |
| TIA / stroke | 1.44 (1.34, 1.55)* | 1.38 (1.25, 1.52)* | 1.52 (1.37, 1.68)* |
| Thrombosis | 2. 42 (2.20, 2.67)* | 2.00 (1.74, 2.30)* | 2.87 (2.49, 3.30)* |
| **First 3 months** | | | |
| Angina / MI | 2.03 (1.82, 2.26)* | 6.09 (3.28, 11.31)* | 2.35 (2.03, 2.72)* |
| AF / flutter / tachycardia | 3.61 (3.40, 3.84)* | 3.03 (2.79, 3.30)* | 4.38 (3.98, 4.81)* |
| Bypass / revascularization | 2.46 (2.12, 2.86)* | 2.79 (1.53, 5.12)* | 2.56 (2.18, 3.01)* |
| All-cause mortality | 4.15 (4.02, 4.28)* | 3.66 (3.51, 3.81)* | 4.95 (4.72, 5.20)* |
| Heart failure | 2.70 (2.51, 2.89)* | 2.60 (2.39, 2.84)* | 2.85 (2.54, 3.21)* |
| Peripheral artery disease | 2.72 (2.44, 3.03)* | 2.43 (2.08, 2.85)* | 3.03 (2.61, 3.52)* |
| TIA / stroke | 2.31 (2.08, 2.56)* | 2.26 (1.94, 2.62)* | 2.37 (2.06, 2.73)* |
| Thrombosis | 3.84 (3.27, 4.51)* | 2.81 (2.34, 3.38)* | 5.85 (3.98, 8.61)* |
| **4 months – end follow-up** | | | |
| Angina / MI | 0.91 (0.81, 1.03) | 0.93 (0.80, 1.09) | 0.90 (0.75, 1.07) |
| AF / flutter / tachycardia | 1.11 (1.05, 1.17)* | 1.05 (0.97, 1.13) | 1.19 (1.10, 1.30)* |
| Bypass / revascularization | 0.99 (0.86, 1.16) | NA | 1.07 (0.90, 1.27) |
| All-cause mortality | 1.29 (1.25, 1.33)* | 1.31 (1.25, 1.36)* | 1.25 (1.19, 1.32)* |
| Heart failure | 1.07 (1.01, 1.14)* | 1.11 (1.03, 1.19)* | 1.01 (0.91, 1.13) |
| Peripheral artery disease | 0.98 (0.86, 1.09) | 0.97 (0.83, 1.14) | 0.99 (0.86, 1.13) |
| TIA / stroke | 0.89 (0.80, 0.99)* | 0.92 (0.80, 1.05) | 0.86 (0.72, 1.02) |
| Thrombosis | 1.26 (1.11, 1.44)* | 1.19 (0.99, 1.44) | 1.34 (1.12, 1.60)* |

Models included 1 covariate, being positive for SARS-CoV-2 or not. Models took into account the matching weights as well as the matching pairs, and cluster-robust standard errors were computed. The model for bypass/revascularization in the last period in females could not be fit. AF: atrial fibrillation; CI: confidence interval; HR: hazard ratio; MI: myocardial infarction; NA: not available, the model could not be fit; TIA: transient ischemic attack. * Denotes statistical significance.

**Supplementary Table S7.** Estimates for the whole period in the sensitivity analysis without matching obtained with accelerated failure time models.

|  | **Crude**  **HR (95% CI)** | **Adjusted**  **HR (95% CI)** |
| --- | --- | --- |
| **All participants** | | |
| Angina / MI | 1.43 (1.33, 1.54)* | 1.54 (1.38, 1.71)* |
| AF / flutter / tachycardia | 2.23 (2.13, 2.33)* | 2.74 (2.56, 2.92)* |
| Bypass / revascularization | 1.75 (1.57, 1.95)* | 1.97 (1.71, 2.27)* |
| All-cause mortality | 3.01 (2.94, 3.09)* | 4.14 (3.96, 4.32)* |
| Heart failure | 1.78 (1.70, 1.88)* | 2.14 (1.97, 2.32)* |
| Peripheral artery disease | 1.77 (1.64, 1.91)* | 1.94 (1.75, 2.16)* |
| TIA / stroke | 1.49 (1.40, 1.59)* | 1.60 (1.46, 1.76)* |
| Thrombosis | 2.49 (2.25, 2.75)* | 2.62 (2.28, 3.01)* |
| **Females** | | |
| Angina / MI | 1.29 (1.16, 1.44)* | 1.38 (1.17, 1.62)* |
| AF / flutter / tachycardia | 1.90 (1.79, 2.02)* | 2.29 (2.08, 2.52)* |
| Bypass / revascularization | 1.42 (1.12, 1.79)* | 1.64 (1.17, 2.29)* |
| All-cause mortality | 2.74 (2.65, 2.84)* | 3.61 (3.39, 3.84)* |
| Heart failure | 1.76 (1.64, 1.88)* | 2.12 (1.91, 2.37)* |
| Peripheral artery disease | 1.66 (1.48, 1.86)* | 1.79 (1.50, 2.12)* |
| TIA / stroke | 2.44 (1.85, 3.23)* | 3.05 (2.09, 4.46)* |
| Thrombosis | 1.41 (1.29, 1.54)* | 1.54 (1.35, 1.75)* |
| **Males** | | |
| Angina / MI | 1.57 (1.41, 1.74)* | 1.70 (1.47, 1.95)* |
| AF / flutter / tachycardia | 2.67 (2.50, 2.84)* | 3.30 (3.01, 3.61)* |
| Bypass / revascularization | 1.92 (1.70, 2.18)* | 2.13 (1.82, 2.50)* |
| All-cause mortality | 3.43 (3.29, 3.56)* | 4.90 (4.62, 5.21)* |
| Heart failure | 1.83 (1.69, 1.99)* | 2.17 (1.92, 2.46)* |
| Peripheral artery disease | 1.89 (1.70, 2.10)* | 2.10 (1.83, 2.41)* |
| TIA / stroke | 1.59 (1.45, 1.75)* | 1.70 (1.49, 1.93)* |
| Thrombosis | 2.87 (2.51, 3.29)* | 3.07 (2.54, 3.70)* |

Crude models included 1 covariate, being positive for SARS-CoV-2 or not. Adjusted models included also the following covariates: age, blood pressure (diastolic and systolic), body mass index, cholesterol (HDL and total), and previous history of cancer, COPD, dementia, hypercholesterolemia, liver and renal insufficiency. Cluster-robust standard errors were computed. AF: atrial fibrillation; CI: confidence interval; HR: hazard ratio; MI: myocardial infarction; TIA: transient ischemic attack. * Denotes statistical significance.

**Supplementary Table S8.** Estimates for the first 3 months in the sensitivity analysis without matching obtained with accelerated failure time models.

|  | **Crude**  **HR (95% CI)** | **Adjusted**  **HR (95% CI)** |
| --- | --- | --- |
| **All participants** | | |
| Angina / MI | 2.10 (1.88, 2.33)* | 2.21 (1.89, 2.57)* |
| AF / flutter / tachycardia | 3.71 (3.48, 3.94)* | 4.77 (4.36, 5.21)* |
| Bypass / revascularization | 2.56 (2.18, 3.00)* | 2.92 (2.37, 3.59)* |
| All-cause mortality | 4.40 (4.26, 4.55)* | 6.28 (5.95, 6.63)* |
| Heart failure | 2.84 (2.64, 3.05)* | 3.54 (3.17, 3.96)* |
| Peripheral artery disease | 2.84 (2.55, 3.17)* | 3.19 (2.76, 3.68)* |
| TIA / stroke | 2.35 (2.12, 2.59)* | 2.54 (2.22, 2.91)* |
| Thrombosis | 3.98 (3.35, 4.72)* | 4.38 (3.45, 5.56)* |
| **Females** | | |
| Angina / MI | 1.02 (0.34, 3.05) | 2.05 (1.50, 2.82)* |
| AF / flutter / tachycardia | 3.11 (2.86, 3.39)* | 3.86 (3.42, 4.34)* |
| Bypass / revascularization | NA | NA |
| All-cause mortality | 3.89 (3.72, 4.06)* | 5.35 (4.95, 5.78)* |
| Heart failure | 2.74 (2.49, 3.01)* | 3.37 (2.90, 3.91)* |
| Peripheral artery disease | 2.53 (2.15, 2.97)* | 2.62 (2.14, 3.22)* |
| TIA / stroke | 2.31 (2.00, 2.68)* | 2.52 (2.06, 3.07)* |
| Thrombosis | 3.08 (2.48, 3.82)* | 3.28 (2.50, 4.30)* |
| **Males** | | |
| Angina / MI | 2.42 (2.09, 2.79)* | 2.33 (1.95, 2.77)* |
| AF / flutter / tachycardia | 4.48 (4.08, 4.91)* | 6.08 (5.28, 7.01)* |
| Bypass / revascularization | 2.73 (2.30, 3.24)* | 3.00 (2.42, 3.71)* |
| All-cause mortality | 5.24 (4.98, 5.51)* | 7.70 (7.11, 8.34)* |
| Heart failure | 3.00 (2.66, 3.38)* | 3.86 (3.24, 4.59)* |
| Peripheral artery disease | 3.19 (2.74, 3.71)* | 3.83 (3.10, 4.72)* |
| TIA / stroke | 2.41 (2.10, 2.76)* | 2.63 (2.19, 3.15)* |
| Thrombosis | 5.11 (3.83, 6.83)* | 6.52 (3.87, 10.98)* |

Crude models included 1 covariate, being positive for SARS-CoV-2 or not. Adjusted models included also the following covariates: age, blood pressure (diastolic and systolic), body mass index, cholesterol (HDL and total), and previous history of cancer, COPD, dementia, hypercholesterolemia, liver and renal insufficiency. Cluster-robust standard errors were computed. The model for bypass/revascularization in females could not be fit. AF: atrial fibrillation; CI: confidence interval; HR: hazard ratio; MI: myocardial infarction; NA: not available, the model or parameter could not be fit. TIA: transient ischemic attack. * Denotes statistical significance.

**Supplementary Table S9.** Estimates for the period 4 months – end of the follow-up in the sensitivity analysis without matching obtained with accelerated failure time models.

|  | **Crude**  **HR (95% CI)** | **Adjusted**  **HR (95% CI)** |
| --- | --- | --- |
| **All participants** | | |
| Angina / MI | 0.94 (0.84, 1.06) | 1.03 (0.87, 1.21) |
| AF / flutter / tachycardia | 1.13 (1.07, 1.19)* | 1.17 (1.07, 1.28)* |
| Bypass / revascularization | 1.02 (0.88, 1.19) | 1.14 (0.94, 1.39) |
| All-cause mortality | 1.35 (1.31, 1.39)* | 1.41 (1.32, 1.50)* |
| Heart failure | 1.11 (1.05, 1.18)* | 1.19 (1.08, 1.32)* |
| Peripheral artery disease | 1.05 (0.92, 1.12) | 1.05 (0.90, 1.22) |
| TIA / stroke | 0.92 (0.82, 1.02) | 0.95 (0.82, 1.11) |
| Thrombosis | 1.30 (1.14, 1.48)* | 1.23 (1.01, 1.49)* |
| **Females** | | |
| Angina / MI | 0.97 (0.83, 1.14) | 1.05 (0.85, 1.29) |
| AF / flutter / tachycardia | 1.07 (0.99, 1.15) | 1.08 (0.95, 1.23) |
| Bypass / revascularization | 0.48 (0.11, 2.13) | 0.46 (0.05, 4.39) |
| All-cause mortality | 2.10 (1.89, 2.31)* | 1.78 (1.46, 2.17)* |
| Heart failure | 1.14 (1.06, 1.23)* | 1.26 (1.11, 1.43)* |
| Peripheral artery disease | 1.00 (0.85, 1.16) | 0.98 (0.75, 1.28) |
| TIA / stroke | 0.93 (0.82, 1.07) | 0.89 (0.71, 1.11) |
| Thrombosis | 1.24 (1.03, 1.50)* | 1.12 (0.85, 1.49) |
| **Males** | | |
| Angina / MI | 0.91 (0.76, 1.09) | 1.00 (0.77, 1.31) |
| AF / flutter / tachycardia | 1.22 (1.12, 1.33)* | 1.29 (1.14, 1.47)* |
| Bypass / revascularization | 1.10 (0.93, 1.31) | 1.24 (1.00, 1.55) |
| All-cause mortality | 1.31 (1.24, 1.39)* | 1.48 (1.35, 1.63)* |
| Heart failure | 1.05 (0.95, 1.17) | 1.09 (0.93, 1.29) |
| Peripheral artery disease | 1.04 (0.91, 1.19) | 1.10 (0.91, 1.32) |
| TIA / stroke | 0.89 (0.75, 1.06) | 1.05 (0.85, 1.28) |
| Thrombosis | 1.36 (1.14, 1.62)* | 1.34 (1.03, 1.75)* |

Crude models included 1 covariate, being positive for SARS-CoV-2 or not. Adjusted models included also the following covariates: age, blood pressure (diastolic and systolic), body mass index, cholesterol (HDL and total), and previous history of cancer, COPD, dementia, hypercholesterolemia, liver and renal insufficiency. Cluster-robust standard errors were computed. AF: atrial fibrillation; CI: confidence interval; HR: hazard ratio; MI: myocardial infarction; TIA: transient ischemic attack. * Denotes statistical significance.

**Supplementary Table S10.** Estimates for the whole period in the sensitivity analysis excluding SARS-CoV-2 positive individuals admitted in the ICU for COVID-19 obtained with accelerated failure time models.

|  | **Crude**  **HR (95% CI)** | **Adjusted**  **HR (95% CI)** |
| --- | --- | --- |
| **All participants** | | |
| Angina / MI | 1.24 (1.15, 1.35)* | 1.36 (1.21, 1.53)* |
| AF / flutter / tachycardia | 1.95 (1.87, 2.05)* | 2.40 (2.24, 2.58)* |
| Bypass / revascularization | 1.66 (1.49, 1.85)* | 1.91 (1.65, 2.21)* |
| All-cause mortality | 2.75 (2.69, 2.82)* | 3.82 (3.65, 3.99)* |
| Heart failure | 1.67 (1.59, 1.76)* | 2.08 (1.91, 2.27)* |
| Peripheral artery disease | 1.69 (1.57, 1.82)* | 1.89 (1.69, 2.11)* |
| TIA / stroke | 1.34 (1.25, 1.45)* | 1.51 (1.36, 1.68)* |
| Thrombosis | 1.90 (1.69, 2.13)* | 2.08 (1.77, 2.45)* |
| **Females** | | |
| Angina / MI | 1.17 (1.04, 1.31)* | 1.33 (1.13, 1.57)* |
| AF / flutter / tachycardia | 1.75 (1.65, 1.86)* | 2.11 (1.91, 2.33)* |
| Bypass / revascularization | 1.39 (1.10, 1.76)* | 1.66 (1.18, 2.34)* |
| All-cause mortality | 2.55 (2.46, 2.63)* | 3.40 (3.19, 3.62)* |
| Heart failure | 1.65 (1.55, 1.73)* | 2.06 (1.84, 2.30)* |
| Peripheral artery disease | 1.62 (1.45, 1.82)* | 1.76 (1.48, 2.10)* |
| TIA / stroke | 1.33 (1.20, 1.47)* | 1.52 (1.31, 1.77)* |
| Thrombosis | 1.76 (1.51, 2.05)* | 1.83 (1.47, 2.29)* |
| **Males** | | |
| Angina / MI | 1.33 (1.18, 1.49)* | 1.42 (1.20, 1.67)* |
| AF / flutter / tachycardia | 2.25 (2.09, 2.41)* | 2.80 (2.52, 3.10)* |
| Bypass / revascularization | 1.82 (1.60, 2.07)* | 2.06 (1.75, 2.42)* |
| All-cause mortality | 3.10 (2.98, 3.22)* | 4.43 (4.16, 4.73)* |
| Heart failure | 1.69 (1.56, 1.84)* | 2.11 (1.85, 2.41)* |
| Peripheral artery disease | 1.78 (1.60, 1.97)* | 2.02 (1.75, 2.34)* |
| TIA / stroke | 1.37 (1.22, 1.53)* | 1.51 (1.30, 1.75)* |
| Thrombosis | 2.07 (1.74, 2.47)* | 2.39 (1.89, 3.03)* |

Crude models included 1 covariate, being positive for SARS-CoV-2 or not. Adjusted models included also the following covariates: age, blood pressure (diastolic and systolic), body mass index, cholesterol (HDL and total), and previous history of cancer, COPD, dementia, hypercholesterolemia, liver and renal insufficiency. Models took into account the matching weights as well as the matching pairs, and cluster-robust standard errors were computed. AF: atrial fibrillation; CI: confidence interval; HR: hazard ratio; MI: myocardial infarction; TIA: transient ischemic attack. * Denotes statistical significance.

**Supplementary Table S11.** Estimates for the first 3 months in the sensitivity analysis excluding SARS-CoV-2 positive individuals admitted in the ICU for COVID-19 obtained with accelerated failure time models.

|  | **Crude**  **HR (95% CI)** | **Adjusted**  **HR (95% CI)** |
| --- | --- | --- |
| **All participants** | | |
| Angina / MI | 1.74 (1.54, 1.96)* | 1.83 (1.53, 2.19)* |
| AF / flutter / tachycardia | 3.23 (3.03, 3.44)* | 4.25 (3.87, 4.66)* |
| Bypass / revascularization | 2.42 (2.07, 2.82)* | 2.79 (2.31, 3.37)* |
| All-cause mortality | 3.97 (3.85, 4.10)* | 5.79 (5.48, 6.12)* |
| Heart failure | 2.60 (2.42, 2.80)* | 3.44 (3.06, 3.86)* |
| Peripheral artery disease | 2.69 (2.41, 3.00)* | 3.09 (2.66, 3.58)* |
| TIA / stroke | 11.99 (8.16, 17.60)* | 16.05 (9.71, 26.51)* |
| Thrombosis | 2.12 (1.90, 2.36)* | 2.41 (2.07, 2.81)* |
| **Females** | | |
| Angina / MI | 4.23 (2.17, 8.26)* | 7.34 (2.05, 26.23)* |
| AF / flutter / tachycardia | 2.86 (2.62, 3.11)* | 3.61 (3.18, 4.09)* |
| Bypass / revascularization | 2.82 (1.54, 5.16)* | NA |
| All-cause mortality | 3.57 (3.42, 3.72)* | 5.00 (4.63, 5.40)* |
| Heart failure | 2.53 (2.31, 2.76)* | 3.29 (2.82, 3.84)* |
| Peripheral artery disease | 2.43 (2.08, 2.85)* | 2.61 (2.11, 3.22)* |
| TIA / stroke | 2.15 (1.84, 2.52)* | 2.46 (1.98, 3.06)* |
| Thrombosis | 2.42 (1.99, 2.94)* | 2.49 (1.96, 3.16)* |
| **Males** | | |
| Angina / MI | 1.98 (1.68, 2.34)* | 1.86 (1.51, 2.29)* |
| AF / flutter / tachycardia | 3.76 (3.41, 4.14)* | 5.19 (4.48, 6.00)* |
| Bypass / revascularization | 2.53 (2.15, 2.99)* | 2.78 (2.29, 3.38)* |
| All-cause mortality | 4.68 (4.45, 4.91)* | 7.02 (6.46, 7.62)* |
| Heart failure | 2.73 (2.42, 3.08)* | 3.70 (3.09, 4.43)* |
| Peripheral artery disease | 2.99 (2.57, 3.49)* | 3.62 (2.93, 4.47)* |
| TIA / stroke | 2.10 (1.80, 2.45)* | 2.40 (1.93, 2.97)* |
| Thrombosis | 4.20 (2.78, 6.35)* | 5.07 (2.91, 8.83)* |

Crude models included 1 covariate, being positive for SARS-CoV-2 or not. Adjusted models included also the following covariates: age, blood pressure (diastolic and systolic), body mass index, cholesterol (HDL and total), and previous history of cancer, COPD, dementia, hypercholesterolemia, liver and renal insufficiency. Models took into account the matching weights as well as the matching pairs, and cluster-robust standard errors were computed. The crude model for bypass/revascularization in females could not be fit. AF: atrial fibrillation; CI: confidence interval; HR: hazard ratio; MI: myocardial infarction; TIA: transient ischemic attack. * Denotes statistical significance.

**Supplementary Table S12.** Estimates for the period 4 months – end of the follow-up in the sensitivity analysis excluding SARS-CoV-2 positive individuals admitted in the ICU for COVID-19 obtained with accelerated failure time models.

|  | **Crude**  **HR (95% CI)** | **Adjusted**  **HR (95% CI)** |
| --- | --- | --- |
| **All participants** | | |
| Angina / MI | 0.90 (0.80, 1.02) | 1.02 (0.86, 1.21) |
| AF / flutter / tachycardia | 1.06 (1.00, 1.12) | 1.09 (0.99, 1.20) |
| Bypass / revascularization | 1.01 (0.87, 1.17) | 1.15 (0.95, 1.40) |
| All-cause mortality | 1.30 (1.26, 1.34)* | 1.39 (1.30, 1.48)* |
| Heart failure | 1.08 (1.02, 1.15)* | 1.19 (1.08, 1.32)* |
| Peripheral artery disease | 0.98 (0.89, 1.09) | 1.04 (0.89, 1.21) |
| TIA / stroke | 0.90 (0.81, 1.00) | 0.95 (0.82, 1.11) |
| Thrombosis | 1.13 (0.98, 1.30) | 1.10 (0.89, 1.36) |
| **Females** | | |
| Angina / MI | 0.94 (0.80, 1.10) | 1.07 (0.86, 1.32) |
| AF / flutter / tachycardia | 1.02 (0.95, 1.10) | 1.01 (0.89, 1.16) |
| Bypass / revascularization | NA | 0.80 (0.45, 1.43) |
| All-cause mortality | 1.31 (1.26, 1.37)* | 1.37 (1.25, 1.49)* |
| Heart failure | 1.11 (1.03, 1.19)* | 1.25 (1.10, 1.41)* |
| Peripheral artery disease | 0.98 (0.84, 1.14) | 0.98 (0.76, 1.28) |
| TIA / stroke | 0.92 (0.81, 1.06) | 0.90 (0.72, 1.12) |
| Thrombosis | 1.17 (0.97, 1.42) | 1.07 (0.79, 1.44) |
| **Males** | | |
| Angina / MI | 0.87 (0.72, 1.05) | 0.97 (0.73, 1.29) |
| AF / flutter / tachycardia | 1.11 (1.01, 1.22)* | 1.19 (1.03, 1.37)* |
| Bypass / revascularization | 1.09 (0.91, 1.30) | 1.26 (1.01, 1.58)* |
| All-cause mortality | 1.27 (1.20, 1.34)* | 1.45 (1.31, 1.59)* |
| Heart failure | 1.02 (0.92, 1.14) | 1.10 (0.92, 1.30) |
| Peripheral artery disease | 1.00 (0.87, 1.15) | 1.09 (0.90, 1.32) |
| TIA / stroke | 0.86 (0.72, 1.03) | 1.04 (0.84, 1.28) |
| Thrombosis | 1.08 (0.87, 1.35) | 1.15 (0.84, 1.56) |

Crude models included 1 covariate, being positive for SARS-CoV-2 or not. Adjusted models included also the following covariates: age, blood pressure (diastolic and systolic), body mass index, cholesterol (HDL and total), and previous history of cancer, COPD, dementia, hypercholesterolemia, liver and renal insufficiency. Models took into account the matching weights as well as the matching pairs, and cluster-robust standard errors were computed. AF: atrial fibrillation; CI: confidence interval; HR: hazard ratio; MI: myocardial infarction; TIA: transient ischemic attack. * Denotes statistical significance.
